# Strengthening the reporting of observational studies in epidemiology using mendelian randomisation (STROBE-MR): explanation and elaboration

**DOI:** 10.1136/bmj.n2233

**Published:** 2021-10-26

**Authors:** Veronika W Skrivankova, Rebecca C Richmond, Benjamin A R Woolf, Neil M Davies, Sonja A Swanson, Tyler J VanderWeele, Nicholas J Timpson, Julian P T Higgins, Niki Dimou, Claudia Langenberg, Elizabeth W Loder, Robert M Golub, Matthias Egger, George Davey Smith, J Brent Richards

**Affiliations:** 1 Institute of Social and Preventive Medicine, University of Bern, Bern, Switzerland; 2 Medical Research Council Integrative Epidemiology Unit, University of Bristol, Bristol, UK; 3 Population Health Sciences, Bristol Medical School, University of Bristol, Bristol, UK; 4 Department of Psychological Science, University of Bristol, Bristol, UK; 5 K G Jebsen Centre for Genetic Epidemiology, Department of Public Health and Nursing, NTNU, Norwegian University of Science and Technology, Trondheim, Norway; 6 Department of Epidemiology, Erasmus MC, Rotterdam, Netherlands; 7 Department of Epidemiology, Harvard T H Chan School of Public Health, Boston, MA, USA; 8 NIHR Bristol Biomedical Research Centre, Bristol, UK; 9 Nutrition and Metabolism Branch, International Agency for Research on Cancer, Lyon, France; 10 Berlin Institute of Health at Charité-Universitätsmedizin Berlin, Berlin, Germany; 11 MRC Epidemiology Unit, University of Cambridge, Cambridge, UK; 12 Harvard Medical School, Boston, MA, USA; 13 *The BMJ*, London, UK; 14 *JAMA*, Chicago, IL, USA; 15 Department of Medicine, Northwestern University Feinberg School of Medicine, Chicago, IL, USA; 16 Centre for Infectious Disease Epidemiology and Research, University of Cape Town, Cape Town, South Africa; 17 Departments of Medicine, Human Genetics, Epidemiology & Biostatistics, Lady Davis Institute, Jewish General Hospital, McGill University, Montreal, QC, Canada; 18 Department of Twin Research and Genetic Epidemiology, King’s College London, University of London, London, UK

## Abstract

Mendelian randomisation (MR) studies allow a better understanding of the causal effects of modifiable exposures on health outcomes, but the published evidence is often hampered by inadequate reporting. Reporting guidelines help authors effectively communicate all critical information about what was done and what was found. STROBE-MR (strengthening the reporting of observational studies in epidemiology using mendelian randomisation) assists authors in reporting their MR research clearly and transparently. Adopting STROBE-MR should help readers, reviewers, and journal editors evaluate the quality of published MR studies. This article explains the 20 items of the STROBE-MR checklist, along with their meaning and rationale, using terms defined in a glossary. Examples of transparent reporting are used for each item to illustrate best practices.

Observational epidemiology often examines the associations between exposures and health outcomes. However, such associations reported in epidemiological studies are often not reliable estimates of causal effects, and can be produced by confounding (that is, by another factor that affects both the outcome and exposure)^1^ ^2^ ^3^ or by other forms of bias. For example, alcohol consumption might be related to many potential confounding factors, including smoking, an unhealthy diet, and limited exercise. In turn, ill health could be related to a reduction or cessation of alcohol consumption, introducing potential bias due to reverse causality, when interest is in studying the effect of alcohol consumption on subsequent health.^4^ ^5^ Several approaches have been developed with the aim of mitigating such biases.^6^ For example, instrumental variable methods rely on an external factor that determines the exposure of interest but is not associated with the outcome other than through its effect on the exposure.^6^ ^7^

Over the past decade, advances in genetic technologies have enabled the identification of thousands of reproducible associations between genetic variation and relevant exposures, traits, and health outcomes. These genetic variations can be used as instrumental variables to analyse the effect of modifiable exposures on diseases through a study method termed mendelian randomisation (MR).^8^ MR studies use genetic variants robustly related to modifiable exposures to understand the influence of the exposure on various health, social, and economic outcomes. Genetic variation is essentially randomly inherited from parents to offspring at conception, and consequently, many factors that confound the association between the exposure and outcome cannot affect the genetic variants. Similarly, genetic variants are generally not influenced by the outcome and therefore, by reverse causation. MR thus provides an opportunity to study the association between exposures and outcomes while reducing potential bias from confounding and reverse causation.^9^

These features make genetic variants suitable candidates as instrumental variables, which can help estimate the causal effects of modifiable exposures on outcomes.^7^ For example, the rs1229984 variant in the alcohol dehydrogenase 1B gene (ADH1B) has been used as an instrument to investigate the causal role of alcohol in cardiovascular disease.^10^ Given these advantages, MR studies have increased in popularity and have begun to inform understanding of disease causation. MR is not limited to studies using genetic variants to generate instrumental variable estimates (box 1, table 1); however, these studies dominate the literature. A glossary of terms commonly used in MR is given in table 2. Additional terms and explanations can be found in a comprehensive open access MR dictionary.^21^

Box 1 **Scope of mendelian randomisation (MR) and the STROBE-MR checklist**

While MR generally uses genetic variation as the instrumental variable, MR is not limited to such studies. Indeed, the term “mendelian randomisation” was introduced in 1991 for investigations of bone marrow transplantation in the treatment of childhood malignancies.^11^ ^12^ The basic notion was that if a child had an HLA compatible sibling, that child was more likely to receive a bone marrow transplant than a child with no compatible sibling. Analysing outcomes according to whether the child has such a sibling (optimally taking the number of siblings into account) is analogous to an intention-to-treat analysis in a randomised clinical trial.^11^ ^12^ Having an HLA compatible sibling (as a matter of chance) could also serve as a genetic instrument for bone marrow transplantation, and so might be used to infer effects of transplantation on cancer outcomes. This approach has continued to be used.^13^ ^14^ ^15^ Initially, MR was defined as the use of germline genetic variation to strengthen causal inference for the influence of modifiable exposures on risk of disease or other outcomes.^16^ This wider definition includes, for example, studies of gene-by-covariate interaction (often with environment as the covariate), for which the interaction cannot be viewed as an instrument for the exposure of interest.^17^ ^18^ Other study designs, such as twin studies, also use the basic principles of mendelian genetics and so can be considered a form of MR. One such example used a male co-twin as an indicator of (on average) higher antenatal testosterone to appraise the effect of testosterone on neurodevelopmental traits.^19^ MR studies range from a simple test of an association between single nucleotide polymorphisms and outcome, which can provide evidence as to whether an exposure affects a disease, to a specific effect estimate from an instrumental variable analysis.

The STROBE-MR (strengthening the reporting of observational studies in epidemiology using mendelian randomisation) guidelines are aimed at the (currently) large majority of MR studies that are implemented within an instrumental variable framework. For MR studies that do not use an instrument for the exposure (such as those of gene-by-environment interaction) or MR studies that use genetic variants in an instrumental variable framework but do not report instrumental variable estimates (such as those of sibling compatibility for transplantation), some items of STROBE-MR will not apply, but the checklist still provides useful guidance. Table 1 gives an overview of study designs addressed and not addressed by STROBE-MR.

**Table 1:** Overview of study designs addressed or not addressed by STROBE-MR checklist

Study types addressed	Study types not addressed
One sample MR studies	Genome wide association studies
Two sample MR studies	Sequencing studies
MR studies after a genome wide association study and reported in the same article	Expression studies
One or two sample MR studies with multiple exposures* or multiple outcomes (or both)	Traditional observational epidemiology studies
Partially applies to MR studies not using genetic variants as instruments for an exposure and those not reporting instrumental variable approaches	

MR=mendelian randomisation; STROBE-MR=strengthening the reporting of observational studies in epidemiology using mendelian randomisation.

* For example, MR studies of circulating protein levels often test the association of hundreds of circulating protein levels, measured on high-throughput assays, with specific outcomes.

**Table 2:** Glossary of commonly used terms in mendelian randomisation (MR)

Term	Explanation
MR	A method that uses genetic variation to strengthen causal inference regarding modifiable exposures (eg, body mass index, alcohol consumption, plasma lipoprotein, time spent in education, C reactive protein level, or serum 25-hydroxyvitamin D) influencing risk of disease or other outcomes. Most MR studies are implemented within an instrumental variable framework, using genetic variants as instrumental variables.
One sample MR	A type of MR study in which one sample of individuals is used to estimate the genetic variant-exposure and genetic variant-outcome associations. This approach requires that the genetic variants, exposures, and outcomes are all measured in the same sample and that individual level data are available on all participants.
Two sample MR	A type of MR study in which the genetic variant-exposure and genetic variant-outcome associations are estimated in different samples and combined using meta-analysis tools. This approach requires summary level statistics of the association of each genetic variant in the two samples. It does not require individual level data.
Bidirectional MR	A type of MR study in which one set of instrumental variables is used to test the effect of the exposure on the outcome and a separate set of instrumental variables is used to test the effect of the outcome on the exposure. This approach allows for a better understanding of the direction of the causal effect.
Instrumental variables	Variables associated with the exposure of interest that are not related to confounders, and that affect the outcome only through the exposure.
Instrumental variable assumptions (core assumptions in MR studies)	Include assumptions for relevance (the genetic variants are associated with the exposure of interest); independence (the genetic variants share no unmeasured cause with the outcome); and exclusion restriction (the genetic variants do not affect the outcome except through their potential effect on the exposure of interest).
Assessment of instrumental variable assumptions	Various tests can assess the plausibility of instrumental variable assumptions (eg, a test of whether potential confounders or pleiotropic mechanisms are associated with the genetic variant; see below for more examples). Only the first assumption (relevance) can be tested conventionally; the validity of the other assumptions cannot be guaranteed. However, tests can provide evidence that they are unlikely to hold (that is, these assumptions cannot be verified, but sometimes can be falsified).
Gene environment equivalence	The notion that differences in an exposure induced by genetic variation will produce the same downstream effects on health outcomes as differences in the exposure produced by environmental influences.
Genetic variant	A variation in the DNA sequence that is found within a population. Typically, a single nucleotide polymorphism (SNP).
Single nucleotide polymorphism (SNP)	A genetic variant in which a single base pair in the DNA varies across the population, at an appreciable frequency. SNPs typically have two alleles (eg, adenine, cytosine, guanine, or thiamine). If the SNP is associated with the trait, then one allele will be associated with a higher value of the trait, the other with a lower value. In MR studies, SNPs are the most common genetic variants used as instrumental variables for a modifiable exposure.
Strand alignment	Strand alignment ensures that the alleles in the exposure GWAS (genome wide association study) and the outcome GWAS are measured on the same DNA strand. An issue could arise when the SNPs are palindromic (that is, guanine/cytosine and adenine/thymine SNPs), which would look the same on both DNA strands. Without ensuring that the exposure and outcome GWAS report the same strand, such SNPs can introduce ambiguity as to whether both the exposure and outcome GWAS are reporting the association with the same effect allele.
Allele score	A single variable produced by combining information from several SNPs that are associated with a trait or phenotype (eg, blood pressure), which can be used to predict the exposure in a MR study. An allele score is sometimes also referred to as genetic risk score, polygenic score, or genetic prediction score.
Linkage disequilibrium	The non-random association of alleles at two or more loci, which normally occurs within a small region of the genome in the general population and is a potential source of bias in MR studies.
r^2^	A measure of the linkage disequilibrium between two genetic loci to quantify their correlation (value of 1 denotes perfect correlation). This measure should not be confused with the R^2^ value (representing the proportion of variation in the exposure variable explained by the genetic variant), which can be used to calculate instrument strength.
Test of instrument strength	Measure of the association between the genetic variant and the exposure. The strength is typically tested using the partial F statistic or the R^2^.
Test for difference	Assessment of the difference between the multivariable adjusted phenotypic association and MR estimates (eg, Hausman test). These tests indicate whether there is any evidence that the estimates differ, over and above estimation error.
Horizontal pleiotropy	When genetic variants affect the outcome via pathways independent of the exposure. This event is a violation of the exclusion restriction assumption and a source of bias in MR studies.
Weak instrument bias	If genetic variants used as instrumental variables are only weakly associated with the exposure of interest, they are said to be “weak instruments,” and then the MR estimates can be biased. Although a partial F statistic is commonly used as an indicator of potential weak instrument bias (when F is <10 in an analysis of one sample), weak instrument bias can still occur at values greater than 10. This rule of thumb is analogous to the false dichotomisation of P values as either significant or not significant at an arbitrary cut-off value such as P=0.05.^20^
Collider bias	Bias that can occur when conditioning on a common effect of the genetic variant and another key variable, such as the outcome. This conditioning can either occur statistically (eg, including a covariate that is caused by both the variant and outcome) or through the study sampling (eg, analysing a sample of patients in hospital, where admission is influenced by the variant and other factors).
Winner’s curse	When the discovery estimates of the SNP-exposure associations tend to be over-estimated, which occurs when the statistically strongest associations—usually using a P value threshold—are selected from the discovery sample.
Data	Can refer to either individual level data, such as measurements of participants’ phenotypes such as body mass index and genetic data, or SNP level phenotype association estimates (summary level data).

**Summary points**

In observational epidemiology, mendelian randomisation (MR) studies provide an opportunity to study the causal association between an exposure and an outcome while reducing the risk of certain biases
Little consensus exists around the reporting of MR studies, and the quality of reporting of these studies has been inconsistent; many MR study reports do not state or examine the various assumptions of MR and report insufficient details on the data sources
STROBE-MR (strengthening the reporting of observational studies in epidemiology using mendelian randomisation), a checklist of 20 reporting items, has been developed for the communication of MR studies
This article explains the rationale of these checklist items and provides examples of transparent reporting
MR study authors, reviewers, and journal editors are encouraged to use STROBE-MR to improve the reporting of these studies

## Strengthening the reporting of MR studies

Despite the growth in MR applications and methods and the increasing relevance of MR findings, little consensus exists around the reporting of MR studies. As a result, the quality of reporting of these studies has been inconsistent. Empirical evidence^22^ ^23^ ^24^ indicates that many reports of MR studies do not clearly state or examine the various assumptions of MR methods and report insufficient details on the data sources, which makes the quality and reliability of the results difficult to evaluate.

The STROBE (strengthening the reporting of observational studies in epidemiology) guidelines for observational research^25^ ^26^ were developed for the three main study designs in epidemiology (cohort, case-control, and cross sectional studies). Some STROBE items are either too general or do not apply to MR studies, while other items relevant to MR studies are missing. To improve reporting MR studies, we developed a separate checklist of items motivated by the STROBE guidelines but explicitly focused on the MR study design, resulting in the STROBE-MR statement (strengthening the reporting of observational studies in epidemiology using mendelian randomisation^27^; table 3). Similar to the STROBE checklist, the STROBE-MR items relate to the title, abstract, introduction, methods, results, and discussion sections of articles. 

**Table 3:** STROBE-MR checklist of recommended items to address in reports of mendelian randomisation studies

Item No	Section	Checklist item
1	**Title and abstract**	Indicate mendelian randomisation (MR) as the study’s design in the title and/or the abstract if that is a main purpose of the study
	**Introduction**	
2	Background	Explain the scientific background and rationale for the reported study. What is the exposure? Is a potential causal association between exposure and outcome plausible? Justify why MR is a helpful method to address the study question
3	Objectives	State specific objectives clearly, including prespecified causal hypotheses (if any). State that MR is a method that, under specific assumptions, intends to estimate causal effects
	**Methods**	
4	Study design and data sources	Present key elements of the study design early in the article. Consider including a table listing sources of data for all phases of the study. For each data source contributing to the analysis, describe the following:
	a)	Setting: Describe the study design and the underlying population, if possible. Describe the setting, locations, and relevant dates, including periods of recruitment, exposure, follow-up, and data collection, when available.
	b)	Participants: Give the eligibility criteria, and the sources and methods of selection of participants. Report the sample size, and whether any power or sample size calculations were carried out prior to the main analysis
	c)	Describe measurement, quality control, and selection of genetic variants
	d)	For each exposure, outcome, and other relevant variables, describe methods of assessment and diagnostic criteria for diseases
	e)	Provide details of ethics committee approval and participant informed consent, if relevant
5	Assumptions	Explicitly state the three core instrumental variable assumptions for the main analysis (relevance, independence, and exclusion restriction), as well assumptions for any additional or sensitivity analysis
6	Statistical methods: main analysis	Describe statistical methods and statistics used
	a)	Describe how quantitative variables were handled in the analyses (that is, scale, units, model)
	b)	Describe how genetic variants were handled in the analyses and, if applicable, how their weights were selected
	c)	Describe the MR estimator (eg, two stage least squares, Wald ratio) and related statistics. Detail the included covariates and, in the case of two sample MR, whether the same covariate set was used for adjustment in the two samples
	d)	Explain how missing data were addressed
	e)	If applicable, indicate how multiple testing was addressed
7	Assessment of assumptions	Describe any methods or prior knowledge used to assess the assumptions or justify their validity
8	Sensitivity analyses and additional analyses	Describe any sensitivity analyses or additional analyses performed (eg, comparison of effect estimates from different approaches, independent replication, bias analytic techniques, validation of instruments, simulations)
9	Software and pre-registration	
	a)	Name statistical software and package(s), including version and settings used
	b)	State whether the study protocol and details were pre-registered (as well as when and where)
	**Results**	
10	Descriptive data	
	a)	Report the numbers of individuals at each stage of included studies and reasons for exclusion. Consider use of a flow diagram
	b)	Report summary statistics for phenotypic exposure(s), outcome(s), and other relevant variables (eg, means, SDs, proportions)
	c)	If the data sources include meta-analyses of previous studies, provide the assessments of heterogeneity across these studies
	d)	For two sample MR: i. Provide justification of the similarity of the genetic variant-exposure associations between the exposure and outcome samples ii. Provide information on the number of individuals who overlap between the exposure and outcome studies
11	Main results	
	a)	Report the associations between genetic variant and exposure, and between genetic variant and outcome, preferably on an interpretable scale
	b)	Report MR estimates of the association between exposure and outcome, and the measures of uncertainty from the MR analysis, on an interpretable scale, such as odds ratio or relative risk per SD difference
	c)	If relevant, consider translating estimates of relative risk into absolute risk for a meaningful time period
	d)	Consider plots to visualise results (eg, forest plot, scatterplot of associations between genetic variants and outcome *v* between genetic variants and exposure)
12	Assessment of assumptions	
	a)	Report the assessment of the validity of the assumptions
	b)	Report any additional statistics (eg, assessments of heterogeneity across genetic variants, such as I^2^, Q statistic, or E value)
13	Sensitivity analyses and additional analyses	
	a)	Report any sensitivity analyses to assess the robustness of the main results to violations of the assumptions
	b)	Report results from other sensitivity analyses or additional analyses
	c)	Report any assessment of direction of causal association (eg, bidirectional MR)
	d)	When relevant, report and compare with estimates from non-MR analyses
	e)	Consider additional plots to visualise results (eg, leave-one-out analyses)
	**Discussion**	
14	Key results	Summarise key results with reference to study objectives
15	Limitations	Discuss limitations of the study, taking into account the validity of the instrumental variable assumptions, other sources of potential bias, and imprecision. Discuss both direction and magnitude of any potential bias and any efforts to address them
16	Interpretation	
	a)	Meaning: Give a cautious overall interpretation of results in the context of their limitations and in comparison with other studies
	b)	Mechanism: Discuss underlying biological mechanisms that could drive a potential causal association between the investigated exposure and the outcome, and whether the gene-environment equivalence assumption is reasonable. Use causal language carefully, clarifying that instrumental variable estimates may provide causal effects only under certain assumptions
	c)	Clinical relevance: Discuss whether the results have clinical or public policy relevance, and to what extent they inform effect sizes of possible interventions
17	Generalisability	Discuss the generalisability of the study results (a) to other populations, (b) across other exposure periods/timings, and (c) across other levels of exposure
	**Other information**	
18	Funding	Describe sources of funding and the role of funders in the present study and, if applicable, sources of funding for the databases and original study or studies on which the present study is based
19	Data and data sharing	Provide the data used to perform all analyses or report where and how the data can be accessed, and reference these sources in the article. Provide the statistical code needed to reproduce the results in the article, or report whether the code is publicly accessible and if so, where
20	Conflicts of interest	All authors should declare all potential conflicts of interest

SD=standard deviation. This checklist is also available separately in web appendix 1.

## Development, scope, and intended use of STROBE-MR

Described in detail elsewhere,^27^ we established this initiative in 2018, following guidance for the development of medical research reporting guidelines.^28^ We invited a group of experts, ranging from MR methodologists and authors of previous reporting guidelines to frequent MR study design users and scientific journal editors to participate in a workshop. The group met for a face-to-face meeting in Bristol, UK, over two days in May 2019 to discuss the empirical evidence on reporting quality of MR studies and draft the checklist items. The draft checklist was published as a preprint in July 2019,^29^ and debated on the preprint platform, social media, and a dedicated session at the 4th International Mendelian Randomisation Conference.^30^ We revised the checklist in the light of the comments received and produced an article presenting the STROBE-MR statement.^27^

The STROBE-MR reporting guidelines are meant to apply to studies that use properties of germline genetic variants to strengthen causal inference regarding possible effects of potentially modifiable exposures on outcomes. The two principal types of MR studies are one sample MR and two sample MR. In a one sample MR study, the associations between the genetic variant and exposure and between the genetic variant and outcome are both measured in the same sample. In a two sample MR study, these two associations are measured in separate samples. MR studies can also use either individual level or summary level data to derive or apply the weights for each single nucleotide polymorphism (SNP). Two sample MR studies are most commonly conducted with summary level data, where the weights are derived from the first sample, which are then applied in the second sample to estimate the gene-outcome association. Summary sample weights for the association of genetic variants with an exposure can also be used in an individual level data analysis of the association of these variants with the outcome. Table 1 (in box 1) outlines the study designs covered and those not covered by the STROBE-MR guidelines.

## Purpose of this article

This explanation and elaboration (E&E) document is intended to complement the STROBE-MR statement.^27^ The format follows that of previous reporting guidelines, such as the STROBE E&E document^26^; it aims to provide readers with a detailed explanation supporting each of the 20 items in the checklist and examples of transparent reporting. Examples of quality reporting for each checklist item have been identified from published MR studies.

This document should be considered as a reference for authors to understand better what is meant by each item in the accompanying checklist. The examples do not necessarily represent the ideal statement for each checklist item. Rather, they highlight the intended issue meant to be covered in each item in the checklist. The boxes and tables in this article contain more theoretical background pertaining to MR study designs and complement recommendations on reporting. Additional guidance on performing MR studies can be found elsewhere.^31^

Some examples were edited by removing citations and sections not related to the reported item. Items are divided into sections: title and abstract (item 1), introduction (items 2-3), methods (items 4-9), results (items 10-13), discussion (items 14-17), and other information (items 18-20; table 3). Some items have subparts that relate to the same topic (eg, item 10d only relates to a two sample MR study design). Additional examples are provided in web appendix 2. We advise authors to address all items in the checklist, even if some information is reported in their supplementary materials because of space restriction.

## Title and abstract (item 1) 

Indicate MR as the study’s design in the title and/or the abstract if that is a main purpose of the study.

### Title

#### Examples

“BMI as a Modifiable Risk Factor for Type 2 Diabetes: Refining and Understanding Causal Estimates Using Mendelian Randomization.”^32^

“Genome Wide Analyses of >200,000 Individuals Identify 58 Loci for Chronic Inflammation and Highlight Pathways that Link Inflammation and Complex Disorders.”^33^

#### Explanation

When MR has played a crucial role in the study design, the term “mendelian randomisation” should be included in the title. In some situations, MR is used as a follow-on analytical technique, when the primary analysis is not MR. In this case, there might be no need to directly include MR in the title but retain focus on the manuscript’s main objectives.

### Abstract

#### Example

“**Importance:** Human genetic studies have indicated that plasma lipoprotein(a) (Lp[a]) is causally associated with the risk of coronary heart disease (CHD), but randomized trials of several therapies that reduce Lp(a) levels by 25% to 35% have not provided any evidence that lowering Lp(a) level reduces CHD risk.

“**Objective:** To estimate the magnitude of the change in plasma Lp(a) levels needed to have the same evidence of an association with CHD risk as a 38.67-mg/dL (ie, 1-mmol/L) change in low-density lipoprotein cholesterol (LDL-C) level, a change that has been shown to produce a clinically meaningful reduction in the risk of CHD.

“**Design, Setting, and Participants:** A Mendelian randomisation analysis was conducted using individual participant data from 5 studies and with external validation using summarised data from 48 studies. Population-based prospective cohort and case-control studies featured 20 793 individuals with CHD and 27 540 controls with individual participant data, whereas summarised data included 62 240 patients with CHD and 127 299 controls. Data were analysed from November 2016 to March 2018.

“**Exposures:** Genetic *LPA* score and plasma Lp(a) mass concentration.

“**Main Outcomes and Measures:** Coronary heart disease.

“**Results:** Of the included study participants, 53% were men, all were of white European ancestry, and the mean age was 57.5 years. The association of genetically predicted Lp(a) with CHD risk was linearly proportional to the absolute change in Lp(a) concentration. A 10-mg/dL lower genetically predicted Lp(a) concentration was associated with a 5.8% lower CHD risk (odds ratio [OR], 0.942; 95% CI, 0.933-0.951; *P*=3×10^−37^), whereas a 10-mg/dL lower genetically predicted LDL-C level estimated using an LDL-C genetic score was associated with a 14.5% lower CHD risk (OR, 0.855; 95% CI, 0.818-0.893; *P*=2×10^−12^). Thus, a 101.5-mg/dL change (95% CI, 71.0-137.0) in Lp(a) concentration had the same association with CHD risk as a 38.67-mg/dL change in LDL-C level. The association of genetically predicted Lp(a) concentration with CHD risk appeared to be independent of changes in LDL-C level owing to genetic variants that mimic the relationship of statins, PCSK9 inhibitors, and ezetimibe with CHD risk.

“**Conclusions and Relevance:** The clinical benefit of lowering Lp(a) is likely to be proportional to the absolute reduction in Lp(a) concentration. Large absolute reductions in Lp(a) of approximately 100 mg/dL may be required to produce a clinically meaningful reduction in the risk of CHD similar in magnitude to what can be achieved by lowering LDL-C level by 38.67 mg/dL (ie, 1 mmol/L).”^34^ (Further examples are available in web appendix 2.)

#### Explanation

The abstract should provide an informative and balanced summary of what was done and what was found. This summary should be presented alongside critical issues in study design, including sources of data, exposures or outcomes, individual versus summary data and would (if possible) include the term “mendelian randomisation” to make the article discoverable as such. Results should be presented in a fully transparent manner and include both point estimates and their error (that is, not only P values) for the range of approaches applied. The word “causal” should be used carefully, because MR only provides estimates intended to inform our understanding of causal associations under specific assumptions. The abstract should be sufficiently detailed to act as a standalone part of the manuscript. When permitted by the journal, structured abstracts can provide clarity and help assure that all relevant information is included. Web appendix 2 includes additional examples of abstracts for one sample, two sample, and embedded MR studies.

## Introduction

### Background (item 2)

Explain the scientific background and rationale for the reported study. What is the exposure? Is causality between exposure and outcome plausible? Justify why MR is a helpful method to answer the study question.

#### Examples

“Epidemiologic studies have reported an increased risk of multiple sclerosis (MS) with earlier age at puberty, particularly among women. However, others failed to replicate this finding. Pubertal timing has complex interactions with weight status, whereby higher childhood adiposity leads to earlier puberty, which in turn is associated with higher adult body mass index (BMI). Because evidence supports a role for increased BMI in MS pathogenesis, at least part of the observed link between pubertal timing and MS might be explained by BMI. Some of the limitations faced by observational studies can be mitigated through instrumental variable methods, in which a variable is used as a proxy for an exposure to explore the effect of that exposure on an outcome. In Mendelian randomisation (MR), genetic variants are used as instrumental variables to test for a causal association between a risk factor and an outcome.”^35^

“Nonlinear relationships exist between risk factors and outcomes across biomedical research. Methods have been developed that help to estimate non-linear relationships between exposures such as BMI and cardiovascular disease . . . We estimated the localized average causal effects of BMI on each risk factor within quintiles of the distribution of BMI after the genetic component is subtracted (the IV [instrumental variable]-free BMI) and performed heterogeneity and trend tests on these values.”^36^

#### Explanation

While some authors have used MR to test the effect of exposures on many different outcomes without prior hypotheses,^37^ most MR studies are designed to assess a specific hypothesis that has arisen from previous studies. When using a specific hypothesis, the rationale for assessing the current hypothesis should be described, including the a priori expectation of the effect size. MR can be used to test causal null hypotheses or estimate point, period, or lifetime effects. The role of MR in assessing the study hypothesis should be delineated to orient readers to what specific gap in the literature can be addressed by applying MR methods to the study hypothesis.

### Objectives (item 3)

State specific objectives clearly, including prespecified causal hypotheses (if any). State that MR is a method that, under specific assumptions, intends to estimate causal effects.

#### Example

“Objective: To evaluate the potential causal association between genetic variants related to elevated serum calcium levels and risk of coronary artery disease (CAD) and myocardial infarction using Mendelian randomization.”^38^

#### Explanation

Authors should clearly state that the study aims to estimate a causal effect of the specified exposure on the specified outcome. This section should define the key exposure(s) and outcome(s) of interest to orient readers, and state the overall study objectives.

## Methods

### Study design and data sources (item 4)

Present key elements of the study design early in the article. Consider including a table listing sources of data for all phases of the study.

#### Example

“Details of the contributing GWAS consortiums are listed in table 1 [see table 4]. The studies were selected for investigating traits related to cardiovascular or metabolic health, having the largest sample sizes, and consisting of the most similar populations while minimising sample overlap. Percentage sample overlap is presented in supplementary table S1. Subjective wellbeing was measured using any items relating to happiness or positive affect and overall life satisfaction. GWAS of each component were meta-analysed to capture subjective wellbeing. For further information on the phenotype definitions and GWAS methods for all traits, see supplementary table S2. All phenotype scores were z scored apart from blood pressure.”^39^

**Table 4:** Description of GWAS consortiums used for each phenotype. Table reproduced with permission from Wootton et al, 2018^39^

Variable	First author (year)	Consortium	Sample size	Population*	Sex*
Subjective wellbeing	Okbay^25^ (2016)	SSGAC	298 420	100% European	Mixed†
Body mass index	Locke^26^ (2015)	GIANT	339 224	95% European	53% female
Waist to hip ratio	Shungin^27^ (2015)	GIANT	210 088	100% European	56% female
Waist circumference	Shungin^27^ (2015)	GIANT	232 101	100% European	55% female
Body fat percentage	Lu^28^ (2016)	Not available	100 716	89% European	48% female
HDL cholesterol	Willer^29^ (2013)	GLGC	92 860	100% European	Mixed†
LDL cholesterol	Willer^29^ (2013)	GLGC	83 198	100% European	Mixed†
Total cholesterol	Willer^29^ (2013)	GLGC	92 260	100% European	Mixed†
Coronary artery disease	Nikpay^30^ (2015)	CARDIoGRAMplusC4D	Cases=60 801; controls=123 504	77% European	Mixed†
Myocardial infarction	Nikpay^30^ (2015)	CARDIoGRAMplusC4D	Cases=43 676; controls=128 199	Mixed†	Mixed†
Diastolic blood pressure	Wain^31^ (2017)	Not available	150 134	100% European	60% female
Systolic blood pressure	Wain^31^ (2017)	Not available	150 134	100% European	60% female

SSGAC=Social Science Genetics Association Consortium; GLGC=Global Lipids Genetics Consortium; GIANT=Genetic Investigation of Anthropometric Traits consortium; HDL=high density lipoprotein; LDL=low density lipoprotein; GWAS=genome wide association study.

* If not reported, percentage sex and European ancestry were calculated from contributing cohort data in the supplementary materials. All GWAS had similar sex ratios and ancestries included. The largest difference was between the consortiums for coronary artery disease and subjective wellbeing, which used 77% and 100% individuals of European ancestry, respectively. If two populations differ, two sample mendelian randomisation can still be used to test for a causal effect, but the magnitude of the effect might not be as precise.^32^

† Information on the sex ratios and ancestry proportions for the whole sample were not reported or not possible to calculate in the CARDIoGRAMplusC4D, GLGC, and SSGAC consortiums.

#### Explanation

As in STROBE,^26^ presenting critical elements of study design early in the article allows readers to orient themselves on the study basics. Authors should clarify whether the MR study used individual level participant data or SNP level summary data, and whether it uses a one sample or two sample MR design. In a two sample MR study, one stage can use summary data and another stage can use individual level data. Some MR studies draw on multiple sources of data (eg, different sources for the ascertainment of the association between the genetic variant and exposure, and for the association between the genetic variant and outcome). Furthermore, sources of data could be from meta-analyses of multiple samples. The general design and data sources should therefore be made clear.

We recommend a table to provide clear documentation of the sources of genetic-variant level information for the MR study (see table 4). For example, the genetic variants used to estimate the exposure could have been ascertained in one study, but the effect size (or weight) of these genetic variants on the exposure taken from a separate study. If so, we recommend reporting both sources of information. The table should be expanded as required. For example, if different MR studies with different outcomes are added to the study, then authors should add additional columns to the table. If additional exposures are studied, then additional rows can be added.

If data were extracted from pre-existing studies, describe how the data were obtained. If data are publicly available, provide a hyperlink to the data source, where possible. If using summary level data, ensure that all of these details are traceable and allow for a qualitative assessment of data sources’ heterogeneity. For each data source contributing to the analysis, describe the following factors in items 4a to 4e.

### Setting (item 4a)

Describe the study design and the underlying population, if possible. Describe the setting, locations, and relevant dates, including periods of recruitment, exposure, follow-up, and data collection, when available.

#### Examples

“This study comprised a meta-analysis of directly genotyped and imputed SNPs from 21 cohorts totalling 42,024 individuals (Table 1). An expanded description of the participating studies is provided in the Text S2.”^40^

“A total of 23 cohorts with genome wide genotyping and fracture data were recruited globally through the GEnetic Factors for OSteoporosis consortium (GEFOS; http://www.gefos.org/). These cohorts were predominantly of European descent and from Europe (n=13), North America (n=8), Australia (n=1), and east Asia (n=1; tables S1A and S2A), and included 20 439 fracture cases and 78 843 controls.”^41^

#### Explanation

Readers need information on the population(s) studied, setting, and locations to assess the context and generalisability of the study results. Exposures such as environmental factors and therapies can change over time. Also, study methods could evolve over time. Knowing when a study took place and over what period participants were recruited and followed up puts the study in historical context, which is essential for interpreting results. Where such information has been described in previous publications, unambiguous reference to these is likely to be sufficient. Providing a description of the ancestry of the participants will help understand potential sources of heterogeneity and generalisability of results. If summary level data from existing studies are used, ensure that details are traceable to allow for a qualitative assessment of any heterogeneity of settings across data sources.

### Participants (item 4b)

Give the eligibility criteria, and the sources and methods of selection of participants. Report the sample size, and whether any power or sample size calculations were carried out before the main analysis.

#### Example 

“The UK Biobank recruited more than 500 000 people aged 37-73 years (99.5% were between 40 and 69 years) from across the country in 2006-10. Participants provided a range of information via questionnaires and interviews (such as demographics, health status, and lifestyle); anthropometric measurements, blood pressure readings, and blood, urine and saliva samples were taken for future analysis. This has been described in more detail elsewhere. We used 120 286 participants of white British descent from the initial UK Biobank dataset, of whom 119 669 had valid genetic data and both BMI and height measures available. We did not include other ethnic groups, because individually they were underpowered.”^42^ (Further examples are available in web appendix 2.)

#### Explanation

Detailed descriptions of the study participants and sampling frame help readers understand the applicability of the results. Authors should provide all the eligibility criteria, participant sources and methods for selecting participants, and methods of follow-up where applicable. Method of recruitment into the study should likewise be described. Where such information has been described in previous publications, unambiguous reference to these is likely to be sufficient. If summary level data from existing studies are used, ensure that details are traceable to allow for a qualitative assessment of participants’ heterogeneity across data sources.

In case-control studies, the choice of cases and controls is crucial to interpreting the results, and the method of their selection has major implications for study validity. In general, controls should reflect the population from which the cases arose.

If sample size or power calculations were conducted before the main analysis, this should also be reported either with the study design or the statistical methods. When planning a study or interpreting the analysis results, information about the sample size is essential. Power calculations provide information about sample sizes needed to obtain appropriate power for desired precision of the effect estimate,^43^ ^44^ and if they are performed, they should be performed before the study was conducted. Statistical power is best ascertained by examination of the 95% confidence intervals of the estimates, since they require fewer assumptions than pre-experiment power calculations.

### Genetic variants (item 4c)

Describe measurement, quality control and selection of genetic variants.

#### Examples

“Genotyping was conducted using the Affymetrix UK Biobank Array. Autosomal analysis was restricted to up to 13,977,204 high-quality Haplotype Reference Consortium imputed variants with a MAF [minor allele frequency] >0.05%, minor allele count >5, info score >0.3, genotype hard call rate >0.95, and Hardy–Weinberg P>1×10^−6^.”^45^

“Genetic markers for various obesity-related risk factors comprised SNPs that were associated with the risk factor of interest (P<5×10^−8^) based on study participants with European ancestry. Correlated SNPs were excluded based on measures of linkage disequilibrium (LD) R2 <0.1 . . . SNPs with ambiguous strand codification (A/T or C/G) were replaced by SNPs in genetic linkage (R2 >0.8) using the proxy snps R package (European populations) (R Project) or were removed from the analyses if the minor allele frequency was higher than 0.4.”^46^

#### Explanation

Providing information on the ascertainment of genotypes and their quality control will enable readers to assess the quality of the genetic variants used in the study. For two sample MR, this information will often require referring to supplementary material presented in previously published articles.

The methods section should provide a clear explanation of the selection and inclusion of specific genetic variants in the analysis. This information would include a description of genetic variants allocated to the exposure of interest and in the case of reverse MR, the genetic variants allocated to the outcome. For each variant, the rsID or base position and chromosome should be disclosed, along with clear reasoning for the variant choice and the reference panel used. The reasoning could include the evidence of association with the exposure or outcome of interest or the characteristics that qualify the specific variant to be used, such as linkage disequilibrium in the case of proxy use. The inclusion of variants in high linkage disequilibrium might not contribute additional information in estimating the causal effect, and could even lead to biased estimates of standard errors if this correlation structure is not accounted for.^47^ Authors should define the threshold used to select independent variants (eg, r^2^), the reference panel, and the population under investigation. However, there are cases in which variants (although in linkage disequilibrium) from a specific gene region with biological relevance regarding the exposure of interest can also be included. In this situation, authors should describe the biological pathways in which these variants are implicated, the r^2^ threshold for inclusion, and which method was used to model the correlation structure.

Studies should also provide estimates of the quality control parameters for the SNPs used in analyses. This information includes the info score (metric of imputation quality), call rate (percentage of individuals with called alleles at a particular SNP), and P value from the Hardy-Weinberg equilibrium test (which could indicate imputation or genotyping problems, population stratification, or non-random mating).

Further information is required to chart the management of genetic variants and harmonisation of datasets in two sample MR analysis. This information includes the conditions used to identify proxy variants in the absence of the same variant being available in both datasets (eg, linkage disequilibrium threshold), the presence or absence and handling of strand alignment, and orientation of effect and non-effect alleles. Other aspects, such as temporal stability of association, population specificity, or biological plausability, could help understand the selected genetic variants’ validity to be used as instrumental variables.

### Assessment and diagnostic criteria for diseases (item 4d)

For each exposure, outcome, and other relevant variables, describe methods of assessment and diagnostic criteria for diseases.

#### Example—continuous exposure or outcome

“Outcomes of the study were WHR [waist-to-hip ratio] (stages 1 and 2b), hip and waist circumference (stage 2a), compartmental body fat masses (stage 3) . . . WHR was defined as the ratio of the circumference of the waist to that of the hip, both of which were estimated in centimeters using a Seca 200-cm tape measure . . . Compartmental fat masses were measured in grams by DEXA, a whole-body, low-intensity x-ray scan that precisely quantifies fat mass in different body regions . . . using a Lunar Prodigy advanced fan beam scanner (GE Healthcare). Participants were scanned by trained operators using standard imaging and positioning protocols. All images were manually processed by one trained researcher, who corrected DEXA demarcations according to a standardised procedure.”^48^ (Further examples are available in web appendix 2.)

#### Explanation

This section provides details on the choice and definition of key exposures, outcomes, and confounders used in the analyses. Where several outcomes or hypothesis-free approaches are used, this information should be clearly indicated, together with any method that accounts for multiple testing. This section will ideally include definitions used in each study (for meta-analyses of different studies) or provide a brief summary with a clear reference if this has previously been described for the study sample. Readers can then consider the case definition’s sensitivity and specificity, and assess the relevance for their question or the generalisability to their population of interest.

### Ethical approval and informed consent (item 4e)

Provide details of ethics committee approval and participant informed consent, if relevant.

#### Example

“Informed consent was obtained from all participants, and study protocols were approved by the local, regional, or institutional ethics committees.”^49^

#### Explanation 

All investigators must ensure that the planning, conduct, and reporting of human research are in accordance with the Helsinki Declaration as revised in 2013.^50^ Authors need to provide information on the approval from the responsible ethics committee and acquisition of the informed consent. This information should also be made available if the data were obtained from publicly available sources or previously conducted studies. Authors should make sure that their study falls into the scope of the original ethics committee approval and does not violate the original agreement.

### Assumptions (item 5)

Explicitly state the three core instrumental variable assumptions for the main analysis (relevance, independence, and exclusion restriction) as well as assumptions for any additional or sensitivity analysis.

#### Examples

“As in any Mendelian randomisation analysis, several assumptions were made, including that the genetic instruments were associated with the risk factor of interest, were independent of potential confounders, and could only affect the outcome through the risk factor and not through alternative pathways (that is, through pleiotropy).”^51^

“Additionally, the slope of MR-Egger regression can provide pleiotropy-corrected causal estimates . . . An important condition of approach is that a SNP’s association with the exposure variable must be independent of its direct effects upon the outcome (previously described as the InSIDE assumption).”^52^

#### Explanation

Explicitly stating the three core instrumental variable assumptions, ideally in the methods sections, can help readers to understand the underlying premises of the MR method, and to allow them to judge their validity. Ideally, the assumptions would be stated in the text using intuitive language specific to the study setting and what they imply in the context of the question being asked. Articulating the assumptions also motivates sensitivity analyses and other additional analyses used to assess the assumptions or the robustness of conclusions to their violations.

**Fig 1:**
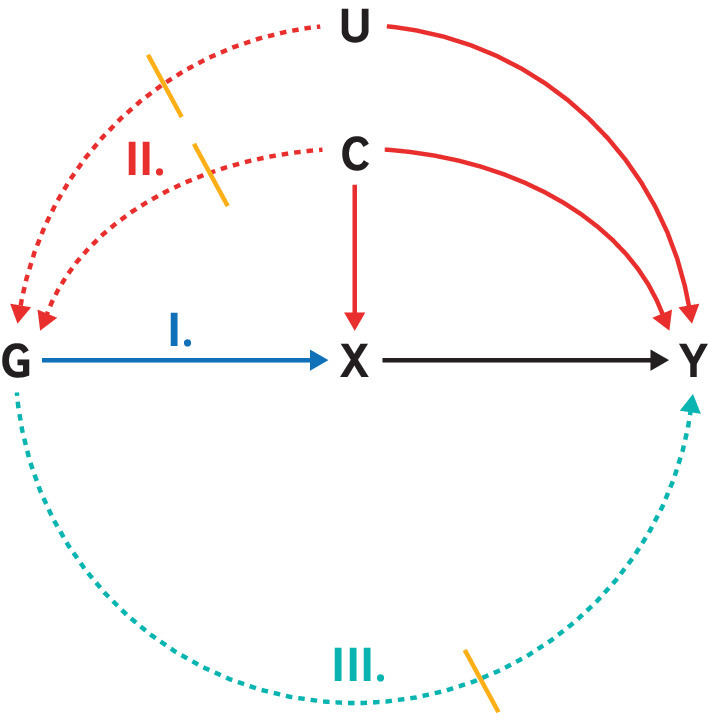
Canonical causal diagram illustrating the assumptions of instrumental variable (IV) analyses. Genetic variant G is used as an instrumental variable (proxy) for exposure X to assess its causal effect on outcome Y. IV assumptions include: I. Relevance: genetic variant G is associated with the exposure of interest X; II. Independence: genetic variant G shares no unmeasured cause with outcome Y; III. Exclusion restriction: genetic variant G does not affect outcome Y except through its potential effect on the exposure of interest X. Solid arrows=causal effects; dashed arrows=causal effects that are specifically prohibited by the IV assumptions. Note that other causal diagrams can be drawn that satisfy the IV assumptions (eg, genetic variant G does not have to directly cause exposure X); likewise, other pathways not drawn might violate the IV assumptions (eg, selection biases can also lead to violation of the independence assumption)

When instrumental variable estimation is used to obtain effect estimates, a fourth assumption should then be stated: typically, an assumption of effect homogeneity^53^ or monotonicity.^54^ In many MR studies, other methods are used to augment traditional instrumental variables estimators (eg, two stage least squares or Wald estimators), and their assumptions should also be stated. For example, MR Egger regression^55^ or weighted median^56^ ^57^ are often used as a supplementary analyses to obtain the estimates when multiple genetic variants are included. Box 2, figure 1, and box 3 give more details on instrumental variable assumptions, common violations, and assessment.

Box 2 **Instrumental variable (IV) assumptions and mendelian randomisation (MR)**

**Core IV estimation assumptions and additional assumptions**

Most MR studies rely on three core IV assumptions (relevance, independence, and exclusion restriction; fig 1, box 3) to carry out testing for causal effects of the exposure on the outcome.^16^ Estimating effect sizes through the instrumental variable approach imposes a fourth assumption, usually homogeneity of effects of the exposure on the outcome.^16^ ^58^ ^59^ ^60^ The homogeneity assumption can also be replaced by imposing a monotonicity assumption that an increase in the number of risk alleles does not lower the likelihood of exposure for any individuals, typically leading to estimating an effect in a subgroup of the study population.^61^

**Violations**

The exclusion restriction is sometimes also referred to as an assumption of no horizontal pleiotropy (box 3), but it can be violated in several other ways (eg, by gene-exposure interaction, by having some form of time varying exposures, by measurement error in the exposure that is related to the instrument or by a multi-component exposure).^59^ Concerns about violations of the independence assumption usually focus on confounding by ancestry (or population stratification). However, it can also be violated by various forms of selection or collider bias, by dynastic effects, or by assortative mating.^62^ ^63^ ^64^ When multiple variants are used in the analysis, these assumptions pertain to each of the variants. Other methods can relax these assumptions, as described below.

**Assumptions for additional analyses**

In many MR studies, instrumental variable methods have been extended in several ways. For example, when multiple genetic variants are used, MR Egger regression,^55^ weighted median,^56^ or weighted mode^57^ are often used as supplementary estimators. MR Egger regression relaxes the exclusion restriction assumption but imposes an InSIDE assumption that the size of the direct effects of the genetic variants on the outcome that do not operate through the exposure are independent of the size of the genetic variants’ effects on the exposure. Additionally, the two sample MR approach assumes that the association between the genetic variants and the exposure is the same in the two samples, which might not hold if samples are selected from different subpopulations (eg, by sex, age, ethnicity).

Box 3 **Assessment of assumptions of mendelian randomisation and sensitivity analyses**

**Relevance**

For the relevance assumption, authors should report how they measured instrument strength. Reporting the F statistic, if individual level data are available, provides several advantages for understanding the risk of weak instrument bias.^65^ The F statistic can also be approximated using summary level data. If the proposed instrument strength is low, reporting should include whether approaches that are robust to weak instruments have been used.

**Independence**

The independence assumption cannot be directly verified, but it can be partially assessed in many research settings. Negative control outcomes or negative control populations can sometimes evaluate the reasonableness of the assumption.^66^ Reporting associations between measured covariates that might confound the variant-outcome association can also prove helpful, particularly if scaled by instrument strength^67^ ^68^ or presented alongside a related bias analytical approach.^69^

**Exclusion restriction**

For the exclusion restriction assumption, MR Egger regression^55^ can be used to detect certain versions of pleiotropy and therefore provide evidence of certain violations of the exclusion restriction. However, the approach depends on an additional assumption (described above) and requires multiple independent variants. Additional approaches to test the exclusion restriction include weighted median^56^ and mode.^57^ The use of negative control outcomes or negative control populations might also allow evaluation of this assumption.^70^ The use of known biological effects of a SNP can also be leveraged to decrease the probability of violation of this assumption.

**Homogeneity**

The homogeneity assumption requires that the exposure has the same effects in everyone, which is not directly verifiable. One possibility for supporting its validity is to determine whether the effect estimate, or even the genetic variants’ effects on the exposure, is the same across subpopulations.^67^ ^71^ Authors can perform stratified or adjusted analyses to relax this assumption if effects with meaningful differences are estimated in different subpopulations.^72^ Furthermore, a global exploration of the homogeneity assumption can examine any differences in variance of a continuous outcome across the genetic instrument; the extent of such differences provides evidence as to extent of violation of the homogeneity assumption.

**Joint falsification strategies**

Some falsification strategies assess assumptions jointly. When using multiple genetic variants as proposed instruments, it is possible to test whether heterogeneity exists across the separate effect estimates (see Test for difference, table 2). Although this test is often interpreted as an assessment of the exclusion restriction, it is jointly testing the exclusion restriction, independence, and homogeneity assumptions. Another relatively straightforward joint test of all assumptions comes from comparing the effect estimate with that obtained by use of a more traditional confounding-adjustment approach.^73^ Assuming that the traditional approach is biased owing to unmeasured confounding, and that the direction of that confounding is suspected, the examination of whether the MR effect estimate aligns with the suspected direction of confounding can support the joint validity of the assumptions underlying the MR effect estimate.

**Sensitivity analyses**

Because several of the estimators using multiple genetic variants rely on different versions of relaxing or adapting the instrumental variable assumptions (eg, MR Egger, median based, or modal based estimators), a comparison of estimates obtained using each of these approaches can help understand the sensitivity of effect estimates to the non-overlapping assumptions of each.^74^ Researchers might also compare MR effect estimates with non-MR estimates, depending on the assumptions underlying alternative methods. Independent replication of MR findings in an independent dataset or with a different study design (eg, one sample *v* two sample MR) is typically advocated to assess the findings’ robustness. Many of the traditional bias analytical techniques in epidemiology can be adapted for MR readily, including the calculations from formulas for understanding the magnitude and direction of confounding bias^59^ ^67^ ^68^ ^69^ ^75^ or for violations of the exclusion restriction.^59^ ^76^ When selection bias is a concern, researchers also frequently conduct simulations to understand the plausible size and direction of bias.^64^ Simulations might also help understand the plausible size and direction of bias induced by assortative mating,^62^ dynastic effects,^9^ ^63^ and time varying effects,^77^ if deemed relevant.

### Statistical methods: main analysis (item 6)

Describe statistical methods and statistics used.

#### Quantitative variables (item 6a)

Describe how quantitative variables were handled in the analyses (that is, scale, units, model).

#### Example

“The effect size for each meta-analysis is reported in the main results as the effect of a one-standard-deviation (1-SD) change in natural-log-transformed 25OHD [25-hydroxyvitamin D] level, since this metric is more interpretable than an arbitrary difference . . . In order to provide a better clinical interpretation of a 1-SD change in natural-log-transformed 25OHD level, we selected three different clinically relevant 25OHD thresholds for vitamin D status (<25 nmol/l for vitamin D deficiency, <50 nmol/l for vitamin D insufficiency, and >75 nmol/l for vitamin D sufficiency).”^78^

#### Explanation

Any transformations made in the quantitative variables (that is, exposure, outcome, or relevant covariates) should be explicitly mentioned, because they affect both interpretation of results and their comparability with other studies. Describing biological knowledge or previous evidence can help justify chosen groupings. When possible, authors should also back-transform estimates to report the units of measurement in common terms to enable future replication of findings. For example, if the effect size is reported in standard deviation change, we suggest reporting the magnitude of the standard deviation for clarification.

#### Genetic variants (item 6b)

Describe how genetic variants were handled in the analyses and, if applicable, how their weights were selected.

#### Example

“We created an allele score from 97 genetic variants previously found to be associated with BMI [body mass index], in a recent GWAS [genome wide association study] meta-analysis by the GIANT consortium. The score was calculated as a sum of the number of BMI-increasing alleles, weighted by the effect size as reported in the GIANT GWAS (reported as a SD [standard deviation] change of BMI per dosage increase such that a higher allele score corresponds to a higher BMI, and was standardised to have a mean of zero and SD of 1).”^79^

#### Explanation

An allele score (also sometimes referred to as genetic risk score, polygenic risk scores, or genetic prediction scores) is a variable that summarises multiple genetic variants in one measure. When many variants are included in the score, bias and coverage probabilities of the instrumental variable estimates are improved compared with estimates from the two stage least squares approach.^80^ Authors should explicitly define the criteria for selecting variants included in the allele score and whether these criteria are based on external data. An allele score could be weighted or unweighted. If weighted, author should clarify whether the weights are derived from the data under analysis or from an independent data source. Authors should also report which genetic model of inheritance is implied in the calculation of genetic variant-exposure and variant-outcome associations (that is, additive or multiplicative). If the weights were derived from the same sample (eg, in a one sample MR), authors should report any efforts to mitigate potential overfitting, typically using methods such as the cross validation or jackknife approaches.

#### Mendelian randomisation estimator (item 6c)

Describe the MR estimator (eg, two stage least squares, Wald ratio) and related statistics. Detail the included covariates and, for two sample MR studies, whether the same covariate set was used for adjustment in the two samples.

#### Example

“Genetic associations with all exposures were taken from a large meta-analysis of GWAS, conducted in adults (n=108,557; mean age, 50.6 years; ~53% men) of European ancestry, without diabetes, adjusted for age, sex, study site and geographic covariates using an additive genetic model . . . Genetic associations with MI [myocardial infarction], angina and heart failure were obtained using logistic regression controlling for age, assay array and 10 principal components in sex-specific analysis and additionally adjusted for sex in the overall analysis, as the adjustment in our previous MR study in the UK Biobank . . . Specifically, we obtained SNP-specific Wald estimates (quotient of genetic association on outcome and genetic association on insulin) and then meta-analysed them using inverse variance weighting (IVW) with multiplicative random effects.”^81^

#### Explanation

The authors should present all the analytical details on the calculation of the instrumental variable estimator. Further clarification on estimating the associated standard errors should also be provided (that is, if this estimator is based on a normal approximation, bootstrapping, or other approaches). Covariates used in the MR analysis should be detailed. For a two sample analysis, authors should state the covariates used to estimate the genetic variant-exposure and genetic variant-outcome associations to assess any differences in the use of covariates between the two associations, which could lead to bias.

#### Missing data (item 6d)

Explain how missing data were addressed.

#### Example

“We conduct our analyses in a Bayesian framework as this lends itself naturally to data imputation. We first introduce a Bayesian complete-case analysis method and then 4 methods for imputing data under the missing-at-random assumption that can be incorporated into the Bayesian model to include subjects with missing data . . . We use cross-sectional baseline data on 3,693 participants who have complete or partial data for C-reactive protein, fibrinogen, and the 3 SNPs. There is missingness in 2.1% of participants for C-reactive protein, 2.4% for fibrinogen, 10.8% for rs1205, 1.9% for rs1130864, and 2.6% for rs1800947.”^82^

#### Explanation

The inclusion of multiple variants with missing data in the estimation of causal effects could decrease precision. Authors should present the percentage of missing data as well as whether any imputation was performed. If imputation was performed the authors should report details of the imputation panel and method of imputation.

#### Multiple testing (item 6e)

If applicable, indicate how multiple testing was addressed.

#### Example

“The significance threshold for all cancer risk and mortality is 0.004 (6 PUFAs times 2 outcomes (risk, mortality) require correcting 0.05 by 12 tests) . . . Given 6 individual cancers were considered, we set a significance threshold of 0.004/36=0.0001.”^83^

#### Explanation

In an MR analysis that involves multiple exposures or multiple outcomes, authors should state whether and how they accounted for multiple testing and provide justification. They should state whether the correction was for the total number of statistically independent exposures or outcomes for all exposures and outcomes. Such a correction could involve reporting false discovery rates, Bonferroni correction, or other techniques, as outlined in the above example.

### Assessment of assumptions (item 7)

Describe any methods or previous knowledge used to assess the assumptions or justify their validity.

#### Examples

“Mendelian randomisation was implemented using the two stage least squares method in the R package *ivpack*. We included age and sex as covariates. To assess the risk of weak instrument bias, we used F tests to determine the strength of association in the first stage regressions between allele score and exposure . . . We used confounding bias plots to assess relative bias in the instrumental variable estimate compared with standard multivariable regression . . . To investigate the degree of bias in the initial causal estimates due to pleiotropic effects, we used two sensitivity analyses (mendelian randomisation-Egger and weighted median mendelian randomisation) . . . Mendelian randomisation-Egger and weighted median methods were implemented using the R package TwoSampleMR.”^84^

“There are reasons for considering MR analysis of a protein drug target to be a distinct category of MR analysis . . . Aside from mRNA expression, differences in protein expression or function are the most proximal consequence of natural genetic variation. This has two consequences: frequently, variants located in and around the encoding gene can be identified with a very substantial effect on protein expression in comparison to other traits; moreover such instruments may also be less prone to violating the ‘no horizontal pleiotropy’ assumption than variants located elsewhere in the genome . . . In the case of MR analysis of proteins, Crick’s ‘Central Dogma’ imposes an order on the direction of information flow from gene to mRNA to encoded protein, which does not extend beyond this to other biological traits that lie more distally in the causal chain that connects genetic variation to disease risk. Finally, *cis-*MR of a protein risk factor greatly reduces the risk of reverse causation, because Crick’s dogma indicates that the pathway gene → encoded protein → disease would always be favoured over the pathway gene → disease → encoded protein, especially given that the gene → encoded protein association is typically derived from population-based (disease-free) samples. Thus, from an MR perspective, proteins are in a privileged position compared with other categories of risk factor and the use of *cis-*MR represents an optimal approach to instrument their causal effect for disease.”^85^

#### Explanation

For each of the assumptions underlying an MR analysis, authors should report any methods used to assess the assumptions or justify their validity. Generally, the subject related background can be used to support the reasonableness of each assumption. While many assumptions cannot be verified, there are methods available to attempt to falsify them. In line with the relevance assumption, the authors can report how they assessed instrument strength. If the proposed instrument strength is low, reporting might include whether approaches that are robust to weak instruments have been used. Although many possible methods are available, some are usable only in certain settings (eg, for dichotomous exposures). Box 3 describes some of the more common and useful approaches, and table 5 lists the commonly used statistics used when examining assumptions and performing sensitivity analyses. The first three core assumptions pertain to any MR analysis with a single instrument; the additional assumptions are needed for instrumental variable estimation. Exclusion restriction is relaxed in sensitivity analyses such as MR Egger regression. These assessments and sensitivity analyses do not represent an exhaustive list of possible strategies, and not all sensitivity analyses are relevant to all MR analyses. For example, F statistics are principally relevant for instrumental variable analyses, because they are approximately equivalent to variance explained in two sample MR approaches based on genome wide association study (GWAS) output. Associations with measured covariates (eg, age, sex, and race or ethnic origin) and effect estimates across subpopulations can be reported with one sample MR studies, but generally not with two sample MR studies, however, GWAS summary statistics are increasingly available for sex and ancestry specific analyses. More comprehensive reviews are provided by Glymour et al^73^ and Labrecque and Swanson.^86^

**Table 5:** Most common instrumental variable assumptions in mendelian randomisation and examples of possible assessments or sensitivity analyses

Assumptions	Examples of possible assessments
Relevance: genetic variants are associated with the exposure of interest	Report F statistic
Independence: genetic variants share no unmeasured cause with the outcome	Report the associations of plausible confounders with both the genetic variant(s) and the outcome; how population stratification has been taken into account (e.g. through principal component adjustment); and unmeasured confounding sensitivity metrics for the variant-outcome association^59^ ^68^ ^69^ ^75^
Exclusion restriction: genetic variants do not affect the outcome except through their potential effect on the exposure of interest	Report results from MR Egger regression slope estimate as well as the intercept and its 95% confidence intervals; and results using negative control outcomes or negative control populations
Homogeneity (two stage least squares): there is a constant causal effect of the exposure of interest on the outcome	Report the instrumental variable effect estimate for different measurable subpopulations (eg, stratified by age, race or ethnic origin, sex, or socioeconomic status); for continuous outcomes, report on variance by level of instrument^72^
InSIDE (MR Egger): associations of genetic variants with the exposure variable must be independent of its direct effects on the outcome	Also report the effect estimates from other estimators that do not require this assumption (eg, median and modal based tests).

### Sensitivity analyses and additional analyses (item 8)

Describe any sensitivity analyses or additional analyses performed (eg, comparison of effect estimates from different approaches, independent replication, bias analytical techniques, validation of instruments, and simulations).

#### Examples

“*Confounding*: We used confounding bias plots to assess relative bias in the instrumental variable estimate compared with standard multivariable regression. Such analyses are designed to quantify the bias present in a mendelian randomisation analysis in a manner analogous to examining the effect of adjusting or not adjusting for a potential confounder in a standard regression analysis. Additionally, in supplementary analyses we included suspected confounding factors as covariates (see supplementary table 4). The confounding variables considered were the first 10 genetic principal components, Townsend deprivation index, birth weight, breast fed, and place of birth (northing and easting coordinates).

“*Horizontal (genetic) pleiotropy*: To investigate the degree of bias in the initial causal estimates due to pleiotropic effects, we used two sensitivity analyses (mendelian randomisation-Egger and weighted median mendelian randomisation). Mendelian randomisation-Egger is not valid for studies in which the instrumental variable-exposure and instrumental variable-outcome associations are calculated in the same sample (as was done for the main analyses in this study). Therefore, we ran the mendelian randomisation-Egger as a split sample analysis, by randomly splitting the sample in half (groups A and B). The supplementary data table shows the associations of the variants and time spent in education and refractive error for each group. Mendelian randomisation-Egger and weighted median methods were implemented using the R package TwoSampleMR (https://github.com/MRCIEU/TwoSampleMR).

“*Measurement error*: To ensure the association between time spent in education and myopia was not an artefact of the non-normal distribution of the variable for age when full time education was completed, we used two alternative methods to recode time spent in education: dichotomisation into age more than 16 years when education was completed and age 16 years or less when education was completed; and excluding those who attended college or university. We compared the results with the original analyses using the continuous variable for age when full time education was completed.”^87^

“Tests of association for individual genetic variants were complemented with gene-based tests of association and S-PrediXcan analysis. The latter was used to identify genes with differential expression levels in cannabis users versus nonusers. We further estimated the genetic correlation of lifetime cannabis use with other traits, including use of other substances and mental health traits, such as schizophrenia. Lastly, we performed bidirectional two-sample Mendelian randomization analysis to examine whether there was evidence for a causal relationship from cannabis use to schizophrenia risk, and from liability to schizophrenia to cannabis use.”^88^ (Further examples are available in web appendix 2.) 

#### Explanation

Sensitivity analyses can test the robustness of effect estimates to plausible violations of the underlying assumptions and help understand the plausible size or direction of bias. Authors should report on any such sensitivity analyses performed. Some common strategies are described in box 3, and further information is available elsewhere.^55^ ^74^ ^86^

### Software and pre-registration (item 9)

#### Statistical software (item 9a)

Name statistical software and package(s), including version and setting used. 

#### Example

“We performed the analysis by using Stata version 14 (StataCorp LP) and R version 3.4.3 (The R Foundation for Statistical Computing). We used the mrrobust package for Stata and the TwoSample MR package for R to facilitate MR analyses.”^89^

#### Explanation 

Statistical methods and software should ideally be described with enough detail to enable a knowledgeable user with access to the original data to verify the reported results. It is preferable to provide the statistical code used in an online repository.

#### Pre-registration (item 9b)

State whether the study protocol and details were pre-registered (as well as when and where).

#### Explanation

Authors should report if a study was pre-registered and provide a link to the study protocol. Examples of pre-registration in MR are rare at present, partly because it poses challenges for secondary data analysis. Potential solutions that protect against researcher bias have been proposed: “The pre-registration can be achieved by pre-specifying the rationale, hypotheses, methods, and analysis plans, and submitting these to either a third-party registry (e.g., the Open Science Framework [OSF]; https://osf.io/), or a journal in the form of a Registered Report.”^90^ Wider adoption of these methods should increase the accuracy, transparency, and robustness of MR studies.

## Results

### Descriptive data (item 10)

#### Number of participants (item 10a) 

Report the numbers of individuals at each stage of included studies and reasons for exclusion. Consider the use of a flow diagram.

#### Example

“UK Biobank recruited 502 664 participants aged 40 to 69 years through 22 assessment centres across the UK . . . All participants completed sociodemographic questionnaires, which included questions on past educational and professional qualifications. In the latter stages of recruitment, an ophthalmic assessment was introduced, and this was completed by approximately 23% of participants . . . In total, 69 798 participants had valid education, refractive error, and genetic data available (fig 1).”^87^

**Fig 2:**
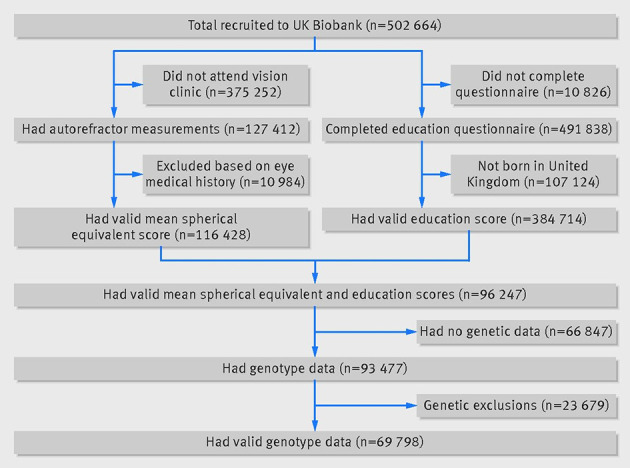
Numbers of participants in UK Biobank who passed validation for mendelian randomisation study. Figure reproduced with permission from Mountjoy et al, 2018^87^

#### Explanation

Information on study participants will help readers understand the target population and assess the validity and generalisability of results. It also provides readers with the information needed to replicate the study and to assess whether the study is likely to show collider bias. If the data sources include individual level data, authors should report information on the participants in the study. Specifically, authors should report the number of individuals at each stage of the study and the reasons why individuals were excluded from further study. Examples of such reasons include loss to follow-up, removal for lack of data, and quality control. Including a STROBE^26^ flowchart for inclusion into the study can quickly provide information about how the study sample was selected. Where possible, report missing values for variables.

#### Summary statistics (item 10b)

Report summary statistics for phenotypic exposure(s), outcome(s), and other relevant variables (eg, means, standard deviations, proportions).

#### Example

“The UK Biobank sample comprised 53.7% women (table 1), and the median age at recruitment was 58.0 years (interquartile range 51.0-63.0). The distribution of adiposity [exposure] and smoking behaviour [outcome] variables in the UK Biobank sample are described in table 1 and table 2 [table 6 and table 7]. As observed in previous studies, current smokers had a lower body mass index than never smokers (−0.22 (95% confidence interval −0.27 to −0.16)). Conversely, former smokers had a higher body mass index than current smokers (1.04 (0.98 to 1.09)).”^51^

**Table 6:** Sample characteristics of body size parameters by smoking and sex categories in UK Biobank. Data are mean (standard deviation). Table reproduced with permission from Carreras-Torres et al, 2018^51^

Body size parameters	Total (n=372 791)	Smoking category				Sex	
		Never (n=203 735)	Former (n=131 537)	Current (n=37 519)		Female (n=200 247)	Male (n=172 544)
Body mass index	27.4 (4.8)	27.1 (4.7)	28.0 (4.7)	27.0 (4.8)		27.0 (5.1)	27.9 (4.2)
Weight (kg)	78.3 (15.9)	77.0 (15.6)	80.5 (16.0)	78.0 (16.3)		71.5 (13.9)	86.2 (14.3)
Height (cm)	168.8 (9.2)	168.3 (9.3)	169.4 (9.1)	169.5 (9.2)		162.7 (6.2)	175.9 (6.7)
Waist circumference (cm)	90.4 (13.5)	88.8 (13.2)	92.6 (13.6)	91.2 (13.5)		84.6 (12.5)	97.1 (11.3)
Body fat percentage (%)	31.4 (8.5)	31.5 (8.6)	31.7 (8.2)	29.9 (8.6)		36.6 (6.9)	25.3 (5.8)

**Table 7:** Sample characteristics of smoking parameters by body mass index and sex categories in UK Biobank ever smokers (current plus former smokers). Data are mean (standard deviation). Table reproduced with permission from Carreras-Torres et al, 2018^51^

Smoking parameters	Total (n=169 056)	Body mass index category						Sex	
		Underweight (<18.5; n=816)		Normal (18.5-25.0; n=49 017)	Overweight (25.0-30.0; n=74 439)	Obese (>30.0; n=44 784)		Female (n=81 091)	Male (n=87 965)
Age started smoking (years)	17.3 (4.2)	17.5 (4.8)		17.6 (4.2)	17.3 (4.2)	17.1 (4.3)		17.8 (4.4)	16.9 (4.0)
No of cigarettes smoked per day									
Ever smokers	18.4 (10.1)	16.6 (10.5)	15.9 (8.6)		18.2 (9.6)	21.1 (11.5)		16.1 (8.2)	20.5 (11.2)
Current smokers*	15.8 (8.4)	16.8 (11.1)	15.0 (8.2)		15.6 (8.1)	17.3 (9.0)		14.2 (7.3)	17.4 (9.2)

* N=37 519 current smokers.

#### Explanation

Information on the distribution of the exposure, outcomes, and other variables helps judge the comparability of groups and generalisability of the findings. Distributions of continuous variables are easily summarised by mean and standard deviation, or by median and percentile range (eg, 25th and 75th percentiles) in case of asymmetrical distribution. Numbers and percentages best describe categorical variables. Readers can assess group differences better if the descriptive statistics are provided for each category separately. Statistical inference regarding differences between groups should be reserved for the main analysis.^26^

In cohort studies in which the outcome is an event, authors should report both the number of events and, if appropriate, the event rate (eg, number of events per person year). A summary measure of follow-up time—such as mean, median, or total follow-up—is also important to understand the period over which events were recorded.

For a time varying outcome, for which time-to-event data are available, the summary measures should be presented over time; a figure could help communicate this. In case-control studies, the summary measures are typically presented separately for cases and controls. A table of continuous exposures or outcomes by categories might also be helpful.^26^

#### Heterogeneity assessment (item 10c)

If the data sources include meta-analyses of previous studies, provide the assessments of heterogeneity across these studies.

#### Example

Table 8 demonstrates the I^2^ test statistic, allowing for an assessment of heterogeneity of the effect of the genetic variants on the outcome across studies. 

**Table 8:** Genome wide significant single nucleotide polymorphisms (SNPs) for fracture. Table reproduced with permission from Trajanoska et al, 2018^41^

Locus	Candidate gene	SNP	Distance to gene (kb)	EA	EAF	Discovery stage*			Replication stage*			Combined*			
						Odds ratio (95% CI)	P		Odds ratio (95% CI)	P		Odds ratio (95% CI)	P	No of fracture cases	I^2^
2p16.2	*SPTBN1*	rs4233949	−23.21	G	0.61	1.03 (1.02 to 1.05)	6.9×10^−5^		1.04 (1.05 to 1.05)	8.9×10^−11^		1.03 (1.02 to 1.04)	2.8×10^−14^	185 057	22.4
3p22.1	*CTNNB1*	rs430727	107.2	T	0.45	1.03 (1.02 to 1.05)	1.0×10^−4^		1.03 (1.02 to 1.04)	1.1×10^−8^		1.03 (1.02 to 1.04)	5.0×10^−12^	185 057	0
6q22.33	*RSPO3*	rs10457487	0	C	0.51	1.06 (1.05 to 1.08)	2.3×10^−15^		1.04 (1.03 to 1.05)	1.7×10^−15^		1.05 (1.04 to 1.06)	4.8×10^−28^	185 057	5
6q25.1	*ESR1*	rs2982570	0	C	0.58	1.05 (1.04 to 1.07)	8.1×10^−12^		1.03 (1.02 to 1.04)	5.2×10^−10^		1.04 (1.03 to 1.05)	4.5×10^−19^	185 057	23
7q31.31	*WNT16*, *CPED1*	rs2908007	−3.25, 24.67	A	0.60	1.08 (1.06 to 1.10)	1.2×10^−20^		1.05 (1.04 to 1.06)	5.6×10^−22^		1.06 (1.05 to 1.07)	2.3×10^−39^	185 055	0
7q21.3	*C7orf76*, *SHFM1*	rs6465508	0, 0	G	0.34	1.05 (1.03 to 1.07)	4.0×10^−9^		1.04 (1.03 to 1.05)	4.1×10^−12^		1.04 (1.03 to 1.05)	2.0×10^−19^	185 056	35
7p14.1	*STARD3NL*	rs6959212	_−_89.01	T	0.34	1.04 (1.02 to 1.06)	6.9×10^−6^		1.02 (1.01 to 1.04)	1.1×10^−5^		1.03 (1.02 to 1.04)	8.8×10^−10^	185 057	15.6
7p12.1	*GRB10*, *COBL*	rs1548607	40.33, −182.4	G	0.32	1.05 (1.03 to 1.07)	3.2×10^−8^		1.02 (1.01 to 1.04)	2.1×10^−4^		1.03 (1.02 to 1.05)	4.7×10^−10^	185 052	40
9q34.11	*FUBP3*	rs7851693	0	G	0.35	1.03 (1.01 to 1.06)	1.3×10^−4^		1.05 (1.06 to 1.06)	4.8×10^−16^		1.04 (1.03 to 1.05)	5.0×10^−19^	185 057	23.5
10q21.1	*MBL2/DKK1*	rs11003047	−90.63	G	0.11	1.09 (1.07 to 1.12)	6.2×10^−12^		1.08 (1.07 to 1.10)	1.4×10^−21^		1.09 (1.07 to 1.10)	9.5×10^−33^	185 057	0
11q13.2	*LRP5*	rs3736228	0	T	0.15	1.05 (1.03 to 1.07)	3.0×10^−5^		1.07 (1.05 to 1.08)	2.8×10^−18^		1.06 (1.05 to 1.08)	1.0×10^−21^	185 056	24.6
14q32.12	*RPS6KA5*	rs1286083	0	T	0.82	1.04 (1.02 to 1.06)	8.8×10^−5^		1.05 (1.04 to 1.07)	3.0×10^−14^		1.05 (1.04 to 1.06)	1.6×10^−17^	185 085	43.3
17q21.31	*SOST*, *DUSP3*, *MEOX1*	rs2741856	−4.26, −16.65, 88.02	G	0.92	1.11 (1.08 to 1.14)	2.4×10^−12^		1.08 (1.06 to 1.11)	5.3×10^−15^		1.10 (1.07 to 1.11)	3.1×10^−25^	184 977	0
18p11.21	*FAM210A*, *RNMT*	rs4635400	0, −7.149	A	0.36	1.06 (1.04 to 1.07)	1.5×10^−12^		1.03 (1.02 to 1.04)	2.7×10^−9^		1.04 (1.03 to 1.05)	1.1×10^−18^	185 057	22
21q22.2	*ETS2*	rs9980072	141.9	G	0.73	1.06 (1.04 to 1.08)	8.4×10^−12^		1.03 (1.01 to 1.04)	1.8×10^−5^		1.04 (1.03 to 1.05)	3.4×10^−13^	185 057	36

EA=effect allele; EAF=effect allele frequency; I^2^=index of heterogeneity.

* Discovery stage (37 857 cases; 227 116 controls); replication stage (147 200 cases; 150 085 controls); combined (185 057 cases; 377 201 controls).

#### Explanation

Evidence on the consistency of the genetic variant’s association with the exposure or outcome helps to understand the degree of heterogeneity of effects. If the estimation is based on a meta-analysis, the number of included studies will also help determine if tests for heterogeneity are properly powered to detect its presence. Presenting 95% confidence intervals along with the I^2^ statistic is recommended.^91^ ^92^

#### Two sample mendelian randomisation (item 10d)

For two sample MR, authors should (1) provide justification of the similarity of the genetic variant-exposure associations between the exposure and outcome samples, and (2) provide information on the number of individuals who were in both samples for the exposure and for the outcome.

#### Example 1

“The genetic variants used for MR were obtained from a GWAS of gallstones conducted in Europeans. A comparison between European and Indian populations with respect to allele frequencies, risk of developing gallstones and gallbladder cancer (GBC) for the genetic variants was made and results are shown in Supplementary Table 1. The allele frequencies between the two populations were generally similar, although with striking differences for some SNPs (e.g. for rs601338, rs1260326, rs174567, rs2469991, rs2290846, where the difference in minor allele frequency was >15%). The risk for developing gallstones and GBC were in broadly the same direction for the SNPs in the Indian population (consistently increased risk for 80% of SNPs in relation to gallstones and 70% SNPs in relation to GBC).”^93^

#### Explanation 1

Two sample MR analyses assume that the SNP-exposure associations are similar in the two samples. For example, the analysis assumes that the two samples are drawn from the same underlying population. However, characteristics such as ethnic origin are not the only relevant factors. Similarity of the two samples can also be violated if, for example, genetic associations were estimated in pre-menopausal versus post-menopausal women, or in a population based sample versus a high risk sample. Where this assumption cannot be made, it should be evaluated by comparing the SNPs’ association with the exposure and the outcome in the two samples, whenever the data are available. If the associations are similar in the two samples, heterogeneity in the associations of the SNPs with the exposure and the outcome is less likely to cause bias. Authors should also provide information on the number of individuals who were in both samples for the exposure and for the outcome.

#### Example 2

“These genome-wide association study estimates were selected from studies that did not include UK Biobank participants, so as to avoid participant overlap, and therefore, in some cases, the genome-wide association study and subsequent instruments differed from the genome-wide association study studies used for the two-sample mendelian randomisation described previously.”^89^

#### Explanation 2

If the authors used the same or similar individuals to estimate the SNP-exposure and the SNP-outcome associations, MR estimates could be biased by a form of the winner’s curse,^94^ ^95^ which occurs when the statistically strongest associations—usually using a P value threshold—are selected from the discovery sample. This bias can be overcome by using entirely separate samples to select SNPs and estimate SNP-outcome associations. The bias is a linear function of the number of individuals included in both samples, so the consequences of a small amount of overlap may not be severe.^94^

### Main results (item 11)

#### Genetic variant associations (item 11a)

Report the associations between genetic variant and exposure, and between genetic variant and outcome, preferably on an interpretable scale.

#### Example

“The BMI allele score created from the 12 BMI-related SNPs showed a positive dose-response association with BMI (per unit increase 0.14% [0.12%–0.16%], *p* = 6.30×10^−62^). The BMI allele score was also associated with 25(OH)D concentrations (per unit increase −0.06% [-0.20% to −0.02%], p=0.004).”^40^

#### Explanation

Reporting the association between the genetic variant and the exposure is required to evaluate the relevance assumption (item 8b). Comparing levels of exposure across the genotype distribution can also indicate monotonicity and linearity of the genetic effect. Reporting on the association between the genetic variant and the outcome is useful because it can provide an initial indication about the possibility of a causal association between the exposure and outcome.

#### Mendelian randomisation estimates (item 11b)

Report MR estimates of the association between exposure and outcome, and the measures of uncertainty from the MR analysis, on an interpretable scale, such as odds ratio or relative risk per standard deviation difference.

#### Example

“The odds ratio of CAD [coronary artery disease] per 1-standard deviation increase in genetically predicted BMI was 1.49 (95% CI [confidence interval] 1.39 to 1.60).”^96^

#### Explanation

If the instrumental variable assumptions are not apparently falsified and are generally supported, or sensitivity analyses are robust to violation of the assumptions (item 8b), then estimates from the MR analysis can be reported in a meaningful manner, preferably on an intuitive scale (eg, relative risk, risk difference). However, if the homogeneity and monotonicity assumptions do not hold, it might be preferable not to report the estimates and outline this situation. Instead, the estimation is replaced by testing for a non-null effect.

#### Calculating absolute risk (item 11c)

If relevant, consider translating estimates of relative risk into absolute risk for a meaningful time period.

#### Example

“LDL [low density lipoprotein] cholesterol lowering alleles at the *NPC1L1* locus were inversely associated with coronary artery disease (OR [odds ratio] for a genetically predicted 1-mmol/L [38.7-mg/dL] reduction in LDL-C of 0.61 [95% CI, 0.42-0.88]; *P*=.008) and directly associated with type 2 diabetes, both individually and collectively (OR for a genetically predicted 1-mmol/L reduction in LDL-C of 2.42 [95% CI, 1.70-3.43], *P*<.001; estimated absolute risk difference, 5.3 incident cases per 1000 person-years for a 1-mmol/L genetically predicted reduction in LDL-C).”^97^

#### Explanation

In some instances, the interpretation of estimates in terms of absolute risks or risk differences rather than relative risk differences might be more clinically meaningful, by taking into account the baseline risk. A measure of absolute risk can provide an estimate of the excess amount of disease that can be attributed to the exposure over a particular period, which can then be used to estimate the absolute benefit of an intervention aimed at reducing levels of the exposure.

#### Visualisation of results (item 11d)

**Fig 3:**
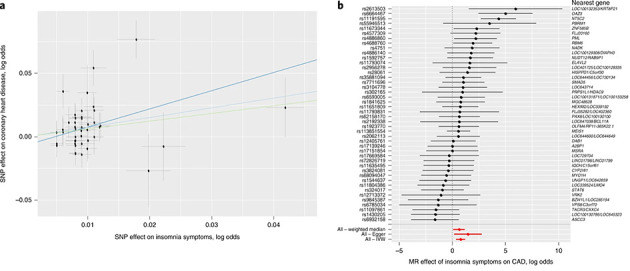
“Causal relationships of insomnia symptoms. (A) Associations between SNPs associated with frequent insomnia symptoms and CAD. Per allele associations with risk plotted against per allele associations with frequent insomnia symptom risk (vertical and horizontal black lines around points show 95% confidence intervals (CI) for each polymorphism) are shown for three different MR association tests. (B) Forest plot showing the estimates of the effect of genetically increased insomnia risk on CAD. Nearest genes are displayed to the right of the plots. Also shown for each SNP is the 95% CI (gray line segment) of the estimate and the IVW MR, MR-Egger, and weighted-median MR results in red. Sample sizes of each GWAS used in the MR analyses are as follows: frequent insomnia symptoms (*n*_cases_=129,270; *n*_controls_=108,352), CAD (*n*_cases_=60,801; *n*_controls_=123,504).”^98^ Figure reproduced with permission from Lane et al, 2019^98^

Consider plots to visualise results (eg, forest plot, scatterplot of associations between genetic variants and outcome versus associations between genetic variants and exposure; see example in fig 3).

#### Example

#### Explanation

Plots can be useful for examining potential violations of the instrumental variable assumptions, especially for the exclusion restriction assumption. Authors should report the associations of the exposure and outcome with the genetic variants individually, which can be presented using a scatterplot or funnel plot.^55^ The scatterplot depicts the association of the genetic effects on the exposure versus the genetic effects on the outcome, with the slope of the line corresponding to the estimated causal effect, with an intercept that is fixed at the origin (except for MR Egger regression; item 8b). A funnel plot, in which causal effect estimates for variants are plotted against their precisions, can be used to perform a visual inspection for asymmetry, which might indicate horizontal pleiotropy.^55^ Forest plots, which plot the causal estimate obtained from each genetic variant, allow for a visual inspection of heterogeneity around the overall causal estimate.^84^

### Assessment of assumptions (item 12)

#### Validity of assumptions (item 12a)

Report the assessment of the validity of the assumptions.

#### Example—relevance assumption

“The myopia allele score explained 4.32% (*F*=3155) of the variance in average mean spherical equivalent refractive error of participants in UK Biobank and the education allele score explained 0.71% (*F*=464) of the variance in time spent in education. We selected these genetic variants to use as instrumental variables because of their robust association with time spent in education and myopia, allowing us to construct strong aggregate instrumental variables for making mendelian randomisation inferences. The large F statistics suggested that these analyses would not be affected by weak instrument bias.”^87^

#### Example—independence assumption

“In tests of the association between the allele scores for time spent in education and myopia with potential confounders, there was evidence that the geographical coordinate, northing (measured northward distance in UK) was negatively associated with time spent in education (β=−1.6e-6, 95% confidence interval −1.8e-6 to −1.5e-6) and positively with refractive error (β=1.2e-6, 9.8e-7 to 1.3e-6). Northing was also associated with the time spent in education (P=7e-5) and myopia (P=6e-3) allele scores (see supplementary table 2). Compared with standard regression, the confounding bias plot suggested that inclusion of the northing variable in the instrumental variable analysis would result in a greater degree of bias for the education allele score but not for the myopia allele score.”^87^

#### Example—exclusion restriction assumption

“MR-Egger, weighted mode, and weighted median methods . . . yielded similar causal estimates in magnitude and direction, such that increasing time spent in education led to a more myopic refractive error (by −0.17 to −0.40 dioptres/y), whereas there was little evidence that a more myopic refractive error led to more time spent in education . . . There was little evidence that the Egger intercept deviated from zero either for more time in education causing refractive error (intercept=0.007, SE=0.006, P=0.2) or refractive error causing more time in education (intercept=−0.002, SE=0.007, P=0.8), indicating that there was little evidence for directional genetic pleiotropy.”^87^

#### Example—homogeneity

“We observed a J shaped relation between genetically predicted BMI and all-cause mortality. The curved shape of the relation was more pronounced in UK Biobank—with higher risk both in underweight participants and in overweight or obese participants. The lowest risk for the overall population was at a BMI of around 22-23 in the HUNT Study and around 25 in UK Biobank.”^99^

#### Explanation

Authors should report the results from assessing the validity of the instrumental variable assumptions, as described under item 7 and box 3. These examples illustrate assessments of these assumptions, but do not represent an exhaustive list of possible assessments or assumptions.

#### Additional statistics (item 12b)

Report any additional statistics (eg, assessments of heterogeneity across genetic variants, such as I^2^, Q statistic, or E value).

#### Examples

“Cochran’s Q and I^2^ statistics were calculated to check for the presence of heterogeneity (dispersion of SNP effects) which can indicate pleiotropy. We found little evidence of heterogeneity for the association between body mass index and wellbeing (see supplementary table S8 for further information).”^39^

“Chen et al. used a single variant in the ALDH2 gene to study the effects of alcohol intake on risk of hypertension. Among males, the variant-hypertension association was an odds ratio of 2.42. The E-value then is 4.27. The E-value for the lower limit of the confidence interval (1.66) is 2.71. As the analysis was conducted in an ethnically homogeneous Asian population, this E-value may be large enough to reasonably conclude that any residual confounding by ancestry is unlikely to explain away the effect.”^69^

#### Explanation

Cochran’s Q and I^2^ statistics can be used to assess evidence of heterogeneity of causal effects estimated by each of the genetic variants.^100^ Evidence of heterogeneity suggests that there is at least one proposed instrument for which at least one of the instrumental variable assumptions fails to hold. The E value^69^ can be used to understand the degree to which unmeasured confounding might explain findings. A large E value could help support that confounding by ancestry is unlikely to explain a non-null effect.

### Sensitivity analyses and additional analyses (item 13)

#### Sensitivity analyses for main results (item 13a)

Report any sensitivity analyses to assess the robustness of the main results to violations of the assumptions.

#### Example

“The fixed-effect inverse-variance weighted and Egger regression estimates suggest an inverse causal effect of CRP [C reactive protein] on CAD risk ​(table 1). However, the corresponding random-effects analyses imply that there is no convincing evidence for a causal effect. Moreover, the simple median estimate is in the opposite direction. This arises because, although the strongest genetic variants have negative causal estimates, the majority of genetic variants have positive causal estimates. The inconsistency of the estimates from different methods indicates that the genome-wide significant variants for CRP are not all valid instrumental variables, and that a causal conclusion based on these variants would be unreliable.”^74^

#### Explanation

Authors should report on, and compare, results obtained from different approaches used to assess the robustness of conclusions to violation of the instrumental variable assumptions, as described in section 7 and box 3. If the results from all the approaches are largely consistent, author can have more confidence in drawing robust conclusions regarding the presence and magnitude of a causal effect.

#### Other analyses (item 13b)

Report results from other sensitivity analyses or additional analyses.

#### Example—independent replication

“The associations of genetically predicted body mass index and waist circumference with risk of being a smoker were replicated in the TAG [Tobacco and Genetics consortium] data (1.19 (1.06 to 1.33) and 1.32 (1.15 to 1.52), respectively.”^51^

#### Example —validation of instruments

“The MR-PRESSO method identified one outlier SNP for heart failure, six outlier SNPs for coronary artery disease, and 11 outlier SNPs for arterial hypertension. Outlier-correction did not materially change the OR estimates for heart failure (1.13; 95% CI 1.08–1.17), coronary artery disease (1.08; 95% CI 1.06–1.10), or arterial hypertension (1.10; 95% CI 1.08–1.12). No outlier SNPs were identified in the MR-PRESSO analysis of the other outcomes.”^101^

#### Example—simulations

“Figure 2 shows that TSLS [two-stage least squares regression methods] is positively biased when there is positive cross‐trait assortative mating on *X* and *Y*. The bias increased proportionally with increasing the degree of assortment. However, both TSLS (2) (i.e., adjusting for parent’s allele scores) and TSLS (3) (i.e., jointly modelling individual’s and parental effects, using nontransmitted allele scores as instruments of parental phenotype) were unbiased with false discovery rates close to 5%.”^62^

#### Explanation

Results of sensitivity analyses or additional analyses, such as independent replication, validation of instruments, and simulation studies, should be presented if they have been performed, as described under item 8.

#### Direction of causality (item 13c)

Report any assessment of direction of causality (eg, bidirectional MR).

#### Example

“The BMI allele score was also associated with 25(OH)D concentrations (per unit increase −0.06%, [−0.10% to −0.02%], p=0.004) while no association with BMI was seen for either the vitamin D synthesis or metabolism allele scores (per allele in synthesis score: 0.01% [−0.17% to 0.20%], p = 0.88, metabolism allele score: 0.17% [−0.02% to 0.35%], p=0.08]).”^40^

#### Explanation

Bidirectional MR can be used to orient the causal direction(s) of effect. This is done using two independent sets of genetic variants related to the exposure and outcome separately, and performing MR analyses to appraise causality in both directions.^102^

#### Compare with non-MR analyses (item 13d)

When relevant, report and compare with estimates from non-MR analyses.

#### Example

“Using the Durbin-Wu-Hausman test for endogeneity, we found weak evidence that the instrumental variable estimate using the time spent in education allele score differed from the observational point estimate (Durbin-Wu-Hausman P=0.06), with the instrumental variable estimate suggesting a larger negative association.”^87^

#### Explanation

Authors should describe important differences between MR estimates and estimates from non-MR analyses. Each study design has different types of biases and could have different degrees of statistical power. Putting the MR results in context will help readers understand if the strengths and weaknesses of MR allow for results that support or contradict previous evidence. In general, causal inference can be presented in a triangulation framework, evaluating the overall body of evidence from several different approaches.^103^ ^104^

#### Additional visualisation of results (item 13e)

Consider additional plots to visualise results (eg, leave-one-out analyses).

#### Example

**Fig 4:**
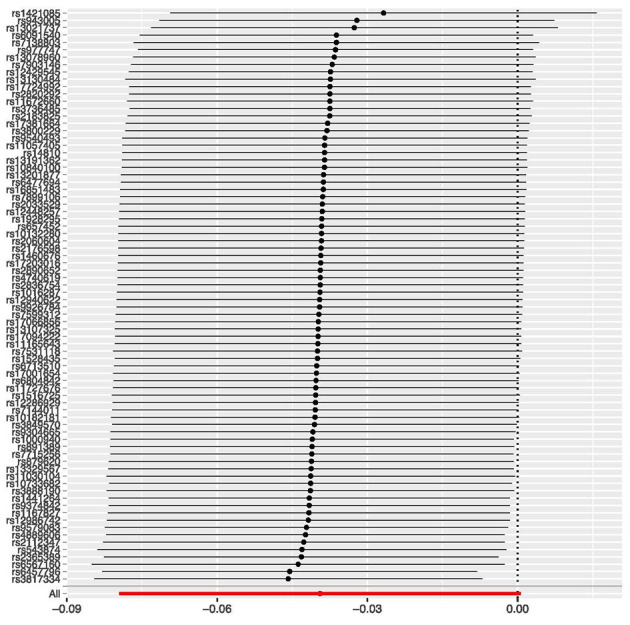
Leave-one-out analysis. Figure reproduced with permission from Wootton et al, 2018^39^

“Leave-one-out analysis: each row represents a two-sample MR analysis of BMI on subjective wellbeing using all of the genome-wide significant SNPs available from Locke et al. except for the SNP listed on the y-axis. The point represents the effect size with that SNP removed and the line represents the standard error. Leave-one-out analysis was conducted using MR Base to identify if any individual SNPs were driving the association between BMI and wellbeing . . . The SNP with the largest contribution to the effect is rs1421085 located on chromosome 16 in the second intron of the FTO (fat mass and obesity associated) gene. FTO has been repeatedly associated with obesity in different populations. However, the biological consequences of intronic FTO SNPs are still unknown. They are currently thought to play a regulatory role in FTO gene expression in the hypothalamus. Although research is not completely certain of the role of FTO, its large effect size and robust association with obesity suggest that this gene has the largest effect in the two-sample MR because of its BMI effect size rather than because of pleiotropic effects.”^39^ (See example in fig 4; further examples are available in web appendix 2.) 

#### Explanation

Additional plots might also aid in the visualisation of results, assessing assumption violation, and detecting potential influential or outlier points. These include the leave-one-out plot,^84^ radial plot,^105^ and plots of each genetic variant against their studentised residuals or Cook’s distance for outlier assessment.^32^

## Discussion

The discussion should look at the important issues pertaining to study interpretation and validity.^106^ Structured discussions can help authors avoid over-interpreting results, and act as a guide for readers.^107^ ^108^

### Key results (item 14)

Summarise key results with reference to study objectives.

#### Example

“Based on comprehensive genetic data from nearly 450 000 individuals, our study provides evidence that differences in body mass index and body fat distribution causally influence different aspects of smoking behaviour, including the risk of individuals taking up smoking, smoking intensity, and smoking cessation. These results highlight the role of obesity in influencing smoking initiation and cessation, which could have implications for public health interventions aiming to reduce the relevance of these important risk factors.”^51^

#### Explanation

The discussion should begin with a summary of the main results and a statement of their importance. This section reminds readers of the study questions and its primary findings, and helps readers assess whether the interpretations that follow are consistent with the results. Good practice would be to keep the summary in the perspective of the main study objectives and focus on the prespecified hypothesis, reporting the estimates of the investigated causal association in the given population.^107^

### Limitations (item 15)

Discuss limitations of the study, taking into account the validity of the instrumental variable assumptions, other sources of potential bias, and imprecision. Discuss both direction and magnitude of any potential biases and your efforts to resolve them.

#### Example

“As in any Mendelian randomisation analysis, several assumptions were made, including that the genetic instruments were associated with the risk factor of interest, were independent of potential confounders, and could only affect the outcome through the risk factor and not through alternative pathways (that is, through pleiotropy). We note that the first assumption was satisfied because robustly associated gene variants were identified from the largest genome wide association study for each obesity parameter. Whether the other two assumptions held was not readily testable, although we conducted thorough sensitivity analyses that did not highlight any obvious violation of these assumptions. Secondly, a potential confounder of our results was population stratification by sociodemographic factors. Indeed, it was previously shown that the genetic instrument for body mass index was associated with various factors related to social class among women, including lower annual household income and level of deprivation. However, no such associations were seen in men. In our study, the associations between the genetic instruments of obesity and individuals taking up smoking and smoking intensity were consistently observed in both men and women, separately, and also when we excluded SNPs that were potentially linked to social deprivation. Therefore, apart from the inverse association between body fat percentage and smoking cessation observed in women only, population stratification by sociodemographic factors would not seem likely to explain those results.”^51^ (Further examples are available in web appendix 2.)

#### Explanation

Authors should address the plausibility of all the instrumental variable assumptions, which is especially important because many of the assumptions are not empirically verifiable. Authors could consider, for example, the possibility that (residual) genotype-phenotype confounders (such as population structure, genetic nurture, or assortative mating) could lead to a violation of the independence assumption. When evaluating a potential violation, authors should identify the sources of a violation that could affect results and discuss the relative importance of different violations, including the likely direction and magnitude of any bias they could induce.

Authors should also discuss the precision of the results. Imprecision can be due to several features of the study design. For example, an instrumental variable estimate’s precision from a meta-analysis of multiple SNPs will usually be greater than that for a single SNP. Suppose SNPs are chosen based on meeting a P value criterion in a discovery GWAS. In that case, authors should consider factors that affect this GWAS’s power to detect SNPs, such as sample size and measurement error. Instrumental variable estimates will also be more precise when estimated from larger datasets, because the standard error for the SNP effect estimates, used to calculate the instrumental variable estimate, will be smaller.

### Interpretation (item 16)

#### Meaning (item 16a)

Give a cautious overall interpretation of results in the context of their limitations and in comparison to other studies.

#### Example

“These Mendelian randomization analyses suggest that the causal effect of CETP (*cholesteryl ester transfer protein*) inhibition on the risk of cardiovascular events appears to be determined by changes in the concentration of apoB-containing lipoproteins rather than changes in LDL-C or HDL-C level.”^109^

#### Explanation

Provide a cautious interpretation of the overall results. When comparing with results from other studies, consider how the results might differ from previous estimates and discuss possible reasons for these differences. Such reasons could include violations of instrumental variable assumptions, imprecision, different estimation methods, and different studied populations. Consider that the overall results should be interpreted in the context of other studies that assessed the study question using different designs, allowing for triangulation of results (see item 13d). When interpreting the effect size, discuss assumptions underlying any extrapolations of effect size and how they may have influenced results.

#### Mechanism (item 16b)

Discuss underlying biological mechanisms that could drive a potential causal association between the investigated exposure and the outcome, and whether the gene-environment equivalence assumption is reasonable. Use causal language carefully, clarifying that instrumental variable estimates might provide causal effects only under certain assumptions.

#### Example

“The association between pubertal timing and weight status is complex and plausibly bidirectional. Increased adiposity in childhood has been linked to earlier pubertal maturation, although this relationship may be nonlinear in boys. Furthermore, several studies report evidence for an association between earlier age at puberty and later obesity. Therefore, we sought to control for both genetically predicted adult and childhood BMI, and we observed a similar magnitude of attenuation in the association between pubertal timing and risk of MS. However, there is a strong association between childhood and adult BMI, which limits the exploration of age-specific effects. Nonetheless, postpubertal rather than childhood obesity is most clearly related to MS susceptibility, making the association between pubertal timing and adult obesity the most likely mediator of the effect of age at puberty on risk of MS. Because it appears that BMI and pubertal timing are in the same causal biological pathway, the association of the selected genetic variants with both exposures represents an example of vertical pleiotropy due to shared biological underpinnings and thus does not bias the MR estimates.”^35^

#### Explanation

While the biological mechanisms that allow genetic variants to be used as instrumental variables are often unknown, the discussion should consider possibilities. Doing so will enable readers to put the MR results in context about possible biological mechanisms, allowing for a better understanding of the plausibility of causal associations.

A common reason why MR estimates do not have a straightforward causal interpretation is because it is unclear whether the gene-environment equivalence assumption (table 2) is plausible (this assumption being that differences in the exposure between genetically defined subgroups of the population are equivalent to differences in the exposure due to an intervention—that is, the MR form of the consistency assumption).^110^ Effects of genetic variants can influence outcomes from conception onwards and in a manner that can be variable and complex. Various components of growth and development could be influenced by the variants, whilst their identification through genetic association studies is often from association with phenotype measured at one time point. The often lifelong influence of genetic variants can be different from the environmental influences investigated in conventional epidemiological studies that are generally experienced at defined stages of later life. Differing effects over the life course do not lessen the potential use of MR estimates. For example, it is possible to separate the effects of body mass index in childhood and adulthood on a variety of health outcomes using different instruments for the two exposure periods.^111^ Conversely, the long term effect of lowered low density lipoprotein (LDL) cholesterol can be estimated from early life onwards by MR and is double the effect estimate derived from randomised trials. This difference probably reflects MR providing an estimate of lifelong effects, whereas trials provide an effect estimate for period of randomisation, which lasts just a few years in cholesterol lowering randomised clinical trials. This difference seen between estimates relating to lifetime differences and shorter term cholesterol lowering is anticipated from the known cumulative effect of lipids on atherosclerotic coronary disease.^112^ The fact that similar estimates are seen in this regard for a wide variety of non-overlapping sets of instruments constructed to match pharmaceutical agents for LDL cholesterol lowering adds robustness to the interpretation.^112^ Time dependency of the effects of instruments is an important issue that should be considered when interpreting MR findings, and is well served by viewing this in the context of gene-environment equivalence.

#### Clinical relevance (item 16c)

Discuss whether the results have clinical or public policy relevance, and to what extent they inform effect sizes of possible interventions.

#### Example

“Although uncertainty remains around the precise function of each of the 162 SNPs, their degree of pleiotropy with cardiac traits, and the mechanisms by which these genetic variants exert their cardioprotective influence, conclusions can still be drawn . . . we note that interventions should be accompanied by careful monitoring for unforeseen side effects, especially in those people who may not thrive when forced into extended educational settings, which may otherwise aggravate health inequalities.”^113^

#### Explanation

Investigators should describe the potential impact of the results on clinical practice or public policy, if any. Because many interventions cannot be tested in randomised clinical trials, MR evidence might help to better understand the possible causal effect of the exposure on the outcome (box 4). Such statements should be made with caution and in the light of evidence from other sources, such as other observational and experimental studies when available. Since clinical and policy interventions might have different effect sizes compared with the genetic variants included in the MR study, extrapolation of this evidence should be clearly described and cautious.

Box 4 **Interpretation of causal effect estimates**

Various considerations are needed when interpreting causal estimates. If the homogeneity assumption is plausible, along with the other assumptions (box 2), then the causal estimate will represent the average causal effect of the exposure on the outcome in the studied population. If the homogeneity assumption cannot be made, but the monotonicity assumption is plausible, then the causal estimate can be used to represent the local average treatment effect.^86^ Caution is especially warranted when interpreting effect estimates with a binary exposure.^114^ In this instance, the homogeneity and monotonicity assumptions are less likely to be plausible. Also, if the exposure is a dichotomisation of a continuous risk factor, this poses a further threat to the violation of the exclusion restriction assumption.^114^ An additional consideration, which is particularly pertinent to the two sample MR setting, is whether the causal effect can truly be attributed to the binary exposure. For example, when two sample MR studies are carried out in exposure samples that contain only a small number of participants who have experienced the exposure in question, it would be misleading to interpret the effects as being those of the exposure itself. Instead, the causal effect estimate should be interpreted as reflecting the effects of the genetic liability to the exposure.^115^ ^116^ Finally, an important component of interpretation is defining the time period.^60^ Usually MR studies are interpreted as a so-called lifetime effect of the exposure, but some settings (eg, MR studies in pregnant women to study prenatal exposures) lend themselves to studies of period effects.

### Generalisability (item 17)

Discuss the generalisability of the study results (a) to other populations, (b) across other exposure periods or timings, and (c) across other levels of exposure.

#### Example

“Our Mendelian randomisation work examined a linear relation between vitamin D levels and fracture risk. We did not test for the possibility of a threshold dependent relation—that is, effects that could be present only at very low levels of vitamin D . . . Finally, the non-significant trend observed for vitamin D towards having increased risk of fracture could be attributed to the selection of healthy people (that is, participants with very low levels of vitamin D and fracture, as well as those who are older, frail, and physically impaired, could have been under-represented in the studies included in the GWAS meta-analyses). Therefore, the vitamin D estimates of the current study cannot be generalised to these groups of older people.”^41^

#### Explanation

The generalisability of a study is the extent to which the study’s results apply to circumstances different from the ones in which the study was conducted.^117^ For example, findings from a cohort of a specific age group collected in the past might not apply to people currently in the same age group.^118^

MR studies can fail to generalise in other ways. For example, because genetic variants might not have a constant effect over the entire life course, authors should consider whether the effect estimate derived in the study would generalise to other exposure periods. For example, if the effect of the exposure on the outcome is time dependent, or only occurs during a critical period, MR estimates could be misleading if used to guide future interventions if they occur outside of this time frame. Likewise, if the effect of an exposure on an outcome is cumulative over many years, MR can overestimate the effect when compared to short term interventions.^60^ ^119^

Also, MR estimates are directly calculated only for the exposure range caused by differences in alleles. Applying MR results, therefore, might not generalise to a wider exposure range. Further, if the MR estimate was derived from a population subgroup, it might not be generalisable beyond that subgroup.

## Other information

### Funding (item 18)

Describe sources of funding and the role of funders in the present study and, if applicable, sources of funding for the databases and original study or studies on which the present study is based.

#### Example

“Funding: The breast cancer genome-wide association analyses were supported by the Government of Canada through Genome Canada and the Canadian Institutes of Health Research, the Ministère de l’Économie, de la Science et de l’Innovation du Québec through Genome Québec and grant PSR-SIIRI-701, the National Institutes of Health (U19 CA148065, X01HG007492), Cancer Research UK (C1287/A10118, C1287/A16563, C1287/A10710), and the European Union (HEALTH-F2-2009-223175 and H2020 633784 and 634935). All studies and funders are listed in Michailidou et al.^25^ RCR, ELA, BMB, CLR, RMM, MM, DAL, and GDS are members of the MRC Integrative Epidemiology Unit at the University of Bristol funded by the Medical Research Council (grant Nos MM_UU_00011/1, MC_UU_00011/2, MC_UU_00011/5, MC_UU_00011/6, and MC_UU_00011/7). RCR is a de Pass VC research fellow at the University of Bristol. This study was supported by the NIHR Biomedical Research Centre at the University Hospitals Bristol NHS Foundation Trust and the University of Bristol. The views expressed in this publication are those of the authors and not necessarily those of the National Health Service, National Institute for Health Research, or Department of Health and Social Care. This work was also supported by Cancer Research UK (grant No C18281/A19169) and the Economic and Social Research Council (grant No ES/N000498/1). SEJ is funded by the Medical Research Council (grant No MR/M005070/1). TMF is supported by the European Research Council (grant No 323195:GLUCOSEGENES-FP7-IDEAS-ERC). MNW is supported by the Wellcome Trust Institutional Strategic Support Award (grant No WT097835MF).”^120^

#### Explanation

The source of research funding can lead to bias or perceptions of bias in the design, conduct, or interpretation of research.^121^ ^122^ This bias is of special concern when research is funded by an entity that has an interest in outcomes that are favourable to its own commercial, academic, or other interests.^123^ Authors should disclose all funding sources and provide detailed information about the role of funders in developing the research question, collecting data, analysing data, selecting investigators, reviewing results, preparing the manuscript, or approving the manuscript for submission or publication. Other sources of influence can include employers, political appointees, and government researchers. Describing the source of funding allows readers to evaluate the work’s credibility and trustworthiness in the light of any potential influence from funders. Authors should disclose funding sources for biobanks or other repositories or databases used in their study because these entities also have commercial interests that might influence research integrity.^124^ ^125^

### Data and data sharing (item 19)

Provide the data used to perform all analyses or report where and how the data can be accessed, and reference these sources in the article. Provide the statistical code needed to reproduce the results in the article, or report whether the code is publicly accessible and if so, where.

#### Example

“Data sharing: The data reported in this paper are available by application directly to the UK Biobank. The genetic associations with the outcomes in the UK Biobank and CARDIoGRAMplusC4D consortium are provided in the supplementary data. Software code in R for implementing the mendelian randomisation analysis, including the principal components analysis, is provided in the supplementary note.”^126^

#### Explanation

Original data are needed by readers or researchers who wish to evaluate or replicate analyses. Many funders and journals encourage or require data sharing, and provide guidance to authors about the content of any explicit data sharing statement required by their journal. Consensus is building that data sharing is “an inseparable part of the research process.”^127^ Ideally, a data sharing plan should be developed when a study is being organised and described in the study protocol and journal publications. The plan and any subsequent data sharing statement in the article should indicate, at a minimum, what data are available (eg, individual participant data, statistical analysis plan, documents related to the study, biobank or other database information) and how the data can be accessed. Contact information for the person or organisation holding the data and a description of the mechanism that will be used to share data should be provided. The statement should also describe any time limits on data availability, processes, and standards applied to requests for data (such as requirements for a research protocol or review of applications by a review board) and, if known, whether a charge is required to obtain the data. If the data are available only via federated analyses, the authors should consider making this clear to readers. When data come from multiple sources, and different conditions apply, consider the use of a table format instead of a text statement.

### Conflicts of interest (item 20)

All authors should declare all potential conflicts of interest.

#### Example

“Competing interests: All authors have completed the International Committee of Medical Journal Editors (ICMJE) uniform disclosure form at www.icmje.org/coi_disclosure.pdf. ARC, DG, TT, JV, REW, GH, RM, SS, SB, GDS, MVH, IT, and AD declare no support from any organisation for the submitted work; no financial relationships with any organisations that might have an interest in the submitted work in the previous three years; no other relationships or activities that could appear to have influenced the submitted work. MRM reports grants from Pfizer and non-financial support from GlaxoSmithKline, outside the submitted work. NMD reports grants from ESRC, grants from MRC, during the conduct of the study; grants from GRAND/Pfizer for unrelated research, outside the submitted work. AET reports grants from Pfizer, outside the submitted work. LDH reports grants from MRC, during the conduct of the study. DW reports grants from NIH, during the conduct of the study.”^89^

#### Explanation

Financial connections between researchers and commercial or other entities and firmly held ideological or intellectual views can lead to bias in the design, conduct, or reporting of study results. When such interests are not disclosed, public trust in the research enterprise is eroded.^128^ According to the International Committee of Medical Journal Editors, “conflict of interest exists when professional judgment concerning a primary interest (such as patients’ welfare or the validity of research) may be influenced by a secondary interest (such as financial gain). Perceptions of conflict of interest are as important as actual conflicts of interest.”^129^ Authors should err on the side of disclosing all matters that might be considered relevant by readers.

## Conclusions

The STROBE-MR reporting guideline proposes a minimum set of items supporting authors to clearly communicate what was planned, what was done, and what was found in an MR study. Similar to the STROBE guidelines^25^ ^26^ for the classical epidemiological study designs—cohort, case-control, and cross sectional studies—the goal is not to be prescriptive of study conduct or limit creativity in the field. Rather, the STROBE-MR guideline is intended to facilitate clear and comprehensive reporting to enable an appraisal of a study’s quality, limitations, and generalisability of findings. The checklist is not intended as a formal tool for assessing the methodological or reporting quality of MR studies, and should not be transformed into a quality scale.^130^ ^131^ STROBE-MR should also not be seen as a formal guideline to design and conduct MR studies. However, some items and text might be useful when designing or conducting an MR study, and this E&E document might be useful to inform methodological decisions, particularly for researchers with less experience in MR research.

We invite readers to comment on STROBE-MR and suggest improvements to the checklist, explanations, and examples. The checklist and E&E document are living documents that we intend to keep up to date on a dedicated website (https://www.strobe-mr.org/). We encourage journals to endorse these guidelines using clear language regarding what they expect from authors and include this information in their instructions to authors. For example, journals could ask authors to submit completed checklists and peer reviewers to use them as part of their review.^28^ The STROBE-MR guidance will be included in the EQUATOR Network website (https://www.equator-network.org/), which provides a comprehensive collection of reporting guidelines and other resources.^132^ In addition, we welcome and wish to be involved in initiatives to translate the checklist and E&E document to other languages.

## Web extra

Extra material supplied by authors

Web appendix 1: STROBE-MR checklist of recommended items to address in reports of Mendelian randomization studies

Web appendix 2: Additional Examples

## Notes

Contributors: ME, GDS, and JBR contributed equally to this manuscript. GDS, NMD, ND, ME, Valentina Gallo, RMG, JPTH, CL, EWL, JBR, RCR, VWS, SAS, NJT, Anne Tybjaerg-Hansen, TJV, BARW, and James Yarmolinsky contributed to the content and elaboration of the STROBE-MR checklist. All authors contributed to the writing of the article and approved of its final version. VWS, RCR, and BARW prepared the first draft of the checklist and discussion material for the workshop. VWS and JBR undertook the practical coordination of STROBE-MR. ME and GDS initiated STROBE-MR and organised the workshop; ME obtained the funding. ME, GDS, and JBR oversaw the project. JBR is the guarantor. The corresponding author attests that all listed authors meet authorship criteria and that no others meeting the criteria have been omitted.
