# Supplementary material for: Strengthening the reporting of observational studies in epidemiology using mendelian randomisation (STROBE-MR): explanation and elaboration
Source: BMJ. 2021 Oct 26;375:n2233. doi: 10.1136/bmj.n2233 (PMC8546498; doi:10.1136/bmj.n2233)
Supplement: Supplementary file 2 — Web appendix 2: Additional Examples [file skrv065837.ww2.pdf]

Strengthening the reporting of observational studies in epidemiology using mendelian randomisation (STROBE-MR): explanation and elaboration

Veronika W Skrivankova, Rebecca C Richmond, Benjamin AR Woolf, Neil M Davies, Sonja A Swanson, Tyler J VanderWeele, Nicholas J Timpson, Julian PT Higgins, Niki Dimou, Claudia Langenberg, Elizabeth W Loder, Robert M Golub, Matthias Egger, George Davey Smith, J Brent Richards

Additional examples

Title and abstract (item 1)

Indicate MR as the study's design in the title and/or the abstract if that is a main purpose of the study.

Example: Abstract - One-sample MR

"Elevated plasma levels of C-reactive protein (CRP), a marker of inflammation, are associated with an increased risk of cancer, but it is unclear whether this association is causal. We examined whether four common single-nucleotide polymorphisms (SNPs) in the CRP gene that are associated with altered plasma CRP levels are causally associated with an increased risk of cancer. The study population included participants in a prospective study (n = 10 215) and a cross-sectional study (n = 36 403) of the adult general population in Denmark, all of whom were genotyped for the CRP SNPs. The association between plasma CRP levels measured by a high-sensitivity turbidimetry assay and the risk of cancer was examined for 8224 participants in the prospective study. The hazard ratio of cancer for a doubling of the plasma CRP level was 1.09 (95% confidence interval [CI] = 1.03 to 1.14). The nine most common genotype combinations of the four CRP SNPs were associated with up to a 72% increase (95% CI = 58% to 87%) in CRP levels but not with an increased risk of cancer. The estimated causal odds ratio for cancer associated with a genetically induced doubling in CRP level was 0.94 (95% CI = 0.81 to 1.08). This finding suggests that elevated CRP levels do not cause cancer." [1]

Example: Abstract - Two sample MR

"**OBJECTIVE** To determine whether body mass index, body fat percentage, and waist circumference influence smoking status and intensity.

DESIGN Mendelian randomisation study.

SETTING UK Biobank, with replication of results from the Tobacco and Genetics (TAG) consortium.

PARTICIPANTS European descent participants from the UK Biobank cohort (n=372 791) and the TAG consortium (n=74 035). MAIN OUTCOME MEASURES Risk of current and past smoking, number of cigarettes smoked per day, age of smoking initiation. RESULTS The Mendelian randomisation analysis indicated that each standard deviation increment in body mass index (4.6) increased the risk of being a smoker (odds ratio 1.18 (95% confidence interval 1.13 to 1.23), P<0.001). This association was replicated in the TAG consortium data (1.19 (1.06 to 1.33), P=0.003). Furthermore, each standard deviation

increment in body mass index was estimated to increase smoking intensity by 0.88 cigarettes per day (95% confidence interval 0.50 to 1.26, P<0.001) in UK Biobank and 1.27 cigarettes per day in the TAG consortium (0.46 to 2.07, P=0.002). Similar results were also seen for body fat percentage and waist circumference in both UK Biobank and the TAG consortium data.

CONCLUSIONS These results strongly suggest that higher adiposity influences smoking behaviour and could have implications for the implementation of public health interventions aiming to reduce the prevalence of these important risk factors.” [2]

Example: Abstract - Embedded MR (as part of a larger analysis)

“**OBJECTIVE:** To identify the genetic determinants of fracture risk and assess the role of 15 clinical risk factors on osteoporotic fracture risk. DeSiGN Meta-analysis of genome wide association studies (GWAS) and a two-sample mendelian randomisation approach.

SETTING: 25 cohorts from Europe, United States, east Asia, and Australia with genome wide genotyping and fracture data.

PARTICIPANTS: A discovery set of 37 857 fracture cases and 227 116 controls; with replication in up to 147 200 fracture cases and 150 085 controls. Fracture cases were defined as individuals (>18 years old) who had fractures at any skeletal site confirmed by medical, radiological, or questionnaire reports. Instrumental variable analyses were performed to estimate effects of 15 selected clinical risk factors for fracture in a two- sample mendelian randomisation framework, using the largest previously published GWAS meta-analysis of each risk factor.

RESULTS: Of 15 fracture associated loci identified, all were also associated with bone mineral density and mapped to genes clustering in pathways known to be critical to bone biology (eg, SOST, WNT16, and ESR1) or novel pathways (FAM210A, GRB10, and ETS2). Mendelian randomisation analyses showed a clear effect of bone mineral density on fracture risk. One standard deviation decrease in genetically determined bone mineral density of the femoral neck was associated with a 55% increase in fracture risk (odds ratio 1.55 (95% confidence interval 1.48 to 1.63; $P=1.5\times10-68$). Hand grip strength was inversely associated with fracture risk, but this result was not significant after multiple testing correction. The remaining clinical risk factors (including vitamin D levels) showed no evidence for an effect on fracture.

CONCLUSIONS: This large scale GWAS meta-analysis for fracture identified 15 genetic determinants of fracture, all of which also influenced bone mineral density. Among the clinical risk factors for fracture assessed, only bone mineral density showed a major causal effect on fracture. Genetic predisposition to lower levels of vitamin D and estimated calcium intake from dairy sources.” [3]

Participants (item 4b)

Give the eligibility criteria, and the sources and methods of selection of participants. Report the sample size, and whether any power or sample size calculations were carried out prior to the main analysis.

Example: “In brief, a total of 1,170 gallbladder cancer cases and 2,525 visitor controls were enrolled into the study. All cases of gallbladder cancer [International Classification of Diseases for Oncology Version 3 (ICD-O-3) site code C23] were microscopically confirmed. Controls were recruited from friends, neighbours, colleagues, in-laws, spouses, and relatives

(other than first-degree relatives) visiting TMH (Mumbai, Maharashtra, India). Controls were frequency matched to cases on age (±10 years), gender, and region. Matching by geographical region (north, north-east, west, central, and south) was conducted using reported place of current residence at the time of enrolment.” [4]

Assessment and diagnostic criteria for diseases (item 4d)

For each exposure, outcome and other relevant variables, describe methods of assessment and diagnostic criteria for diseases.

Example: Categorical exposure or outcome

“The outcome of the study was prevalent type 2 diabetes, defined consistent with validated algorithms developed for UK Biobank. (Eastwood et al Plos One 2016) Participants were classified as cases if they met the following 2 criteria: (1) self-reported type 2 diabetes diagnosis or self-reported diabetes medication at nurse interview or at digital questionnaire, or electronic health record consistent with type 2 diabetes (International Statistical Classification of Diseases and Related Health Problems Tenth Revision code E11); and (2) age at diagnosis older than 36 years or use of oral antidiabetic medications (to exclude likely type 1 diabetes cases). Controls were participants who (1) did not self-report a diagnosis of diabetes of any type, (2) did not take any diabetes medications, and (3) did not have an electronic health record of diabetes of any type.” [5]

Example: All-cause and cause-specific mortality

“Data from death certificates were sent to UK Biobank on a quarterly basis provided by the National Health Service (NHS) Information Centre for participants from England and Wales and by NHS Central Register, Scotland for participants from Scotland. More detailed information on mortality are available at http://biobank.ctsu.ox.ac.uk/crystal/refer.cgi?id=115559. The death certificates include the disease or condition stated to be the underlying cause of death, as well as other conditions, diseases, injuries or events contributing to death but not related to the disease or condition causing it. Data were provided as date of death (DoD), an integer value for age of death (AoD) and underlying (primary) cause of death in International Classification of Diseases (ICD)-10 codes for all deaths that occurred between the 10/05/2006 and 16/02/2016. Rather than using the integer value of AoD from the death certificate, a more precise measure of AoD was derived by adding the time interval between date of initial assessment and DoD (in days) to the participant’s age at initial assessment. All participants who were not recorded as dead by the 16th of February 2016 were assumed to still be alive. The ICD-10 codes were categorised into all-cause and cause-specific mortality as presented in Table S1a. As of August 2017 (date of extraction for all data), there were 14,417 total deaths in the entire UK Biobank dataset that had occurred up to 16th of February 2016 (Table S1a for the whole sample and Table S1b for males and females), which remains the most updated data on mortality.

For the purposes of this study, the primary outcomes of focus were as follows: all-cause mortality and mortality from all cardiovascular diseases and those specifically due to coronary heart disease, stroke, aortic aneurysm and any other cardiovascular diseases; overall cancer and those specifically due to cancers of the lung, colorectum, prostate (men only), breast cancer (women only, separated into pre- and post-menopausal occurrences), pancreas, ovaries (women only), endometrium (women only), stomach, oesophagus, skin

(malignant melanoma), kidney, bladder, brain, lymphatic system and all other cancers; and external causes.” [6]

Descriptive data: Number of participants (item 10a)

Report the numbers of individuals at each stage of included studies and reasons for exclusion. Consider the use of a flow diagram.

Example: ”UK Biobank is a prospective cohort that recruited more than 500 000 men and women aged 40- 96 years between 2006 and 2010, and collected anthropometric, health, and lifestyle data, as well as biological samples. Of 487 409 individuals who were genotyped in UK Biobank, we used data for 372 791 European descent participants with valid adiposity and smoking behaviour measures at recruitment. European background was genetically assessed through principal component analyses of data from genome wide association studies. Sample quality control steps are given in the supplementary methods.” [2]

Example: “Supplementary figure 1 shows the exclusion criteria for the main UK Biobank analyses, and supplementary figure 2 shows the exclusion criteria for the genome-wide association studies carried out for systolic blood pressure and smoking. White British participants were defined by using both self-reported questionnaire data and similar genetic ancestry to the European ancestry principal components computed from the 1000 genomes project.” [7]

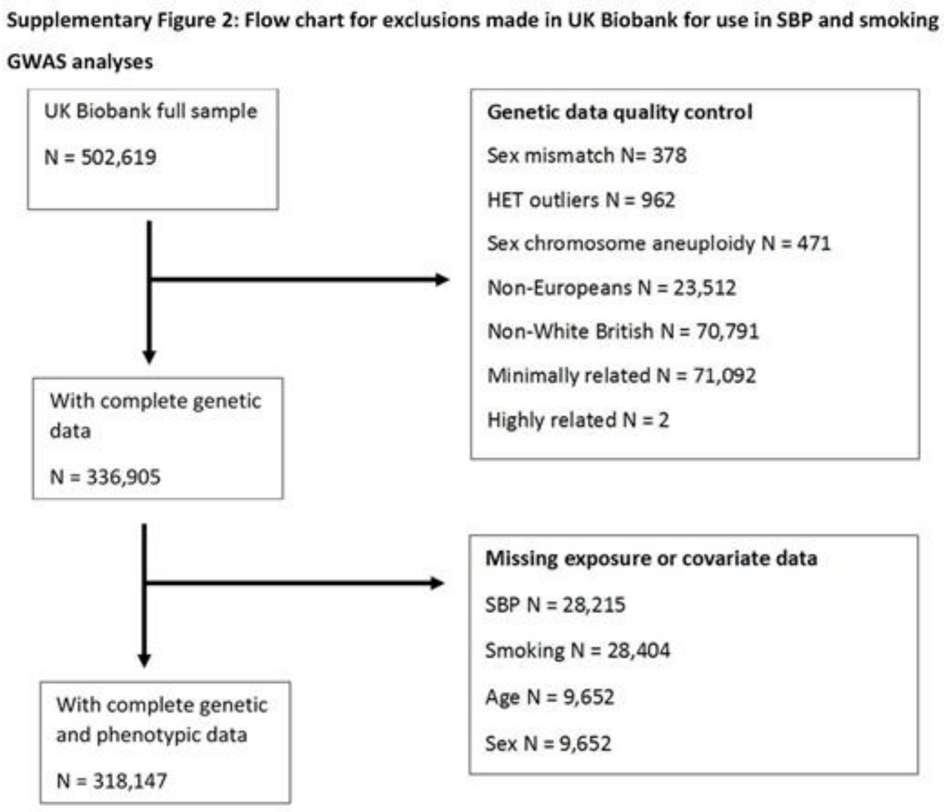

Figure reproduced with permission from Carter et al., 2019.[7]

Additional visualisation of results (item 13e)

Consider additional plots to visualize results (e.g., leave-one-out analyses).

Example: Radial plot

“Figure 6. Radial plots of the blood-pressure data produced using the RadialMR package. Top: Only the IVW estimate shown, Radial lines joining each data point back to the origin. Bottom: Radial MR-Egger and IVW model fits shown.” [8]

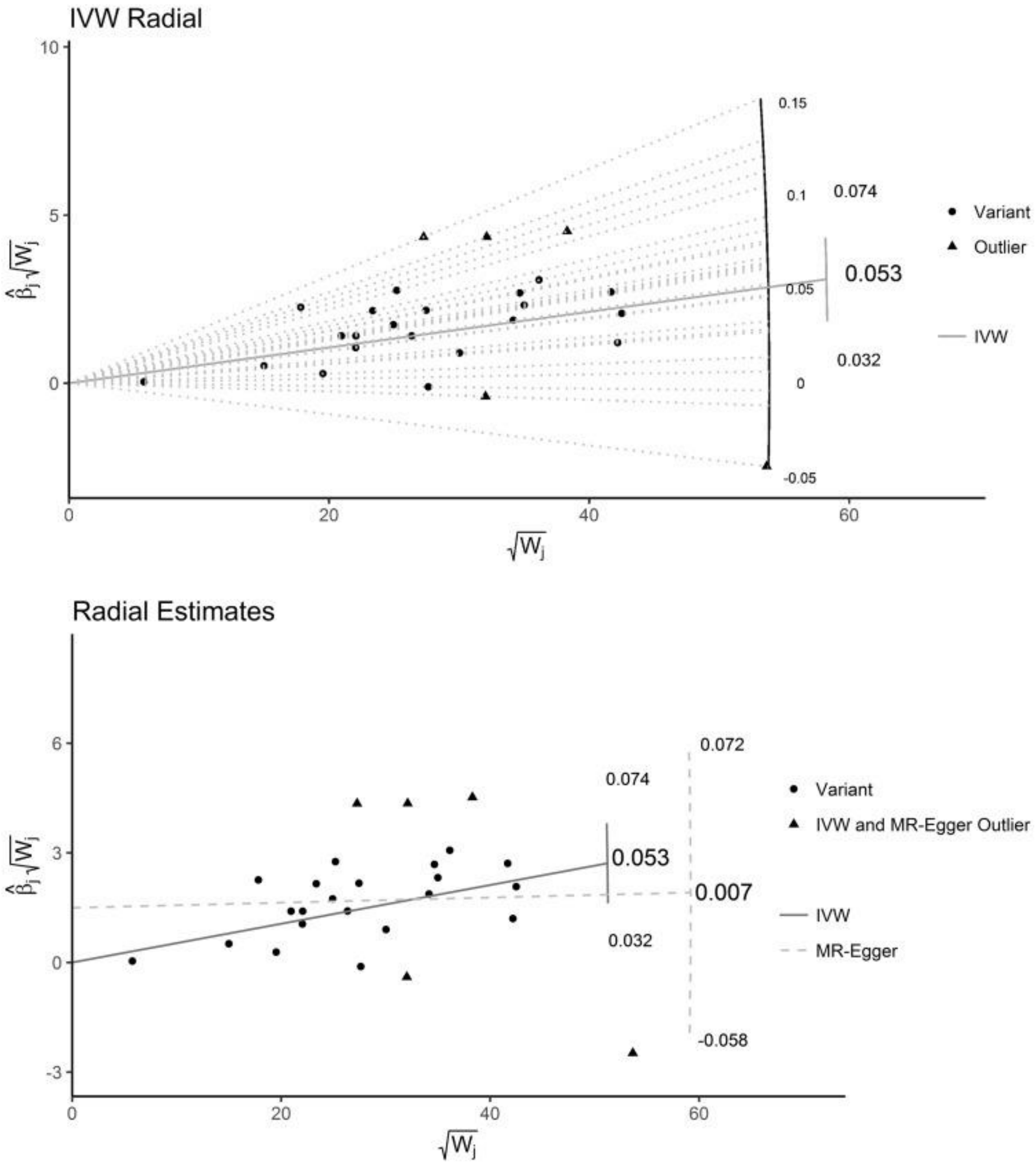

Figure reproduced with permission from Bowden et al., 2018.[8]

Example: Outlier analysis – Studentised residuals

“Figure S1A - Studentised residuals applied to the IVW method.” [9]

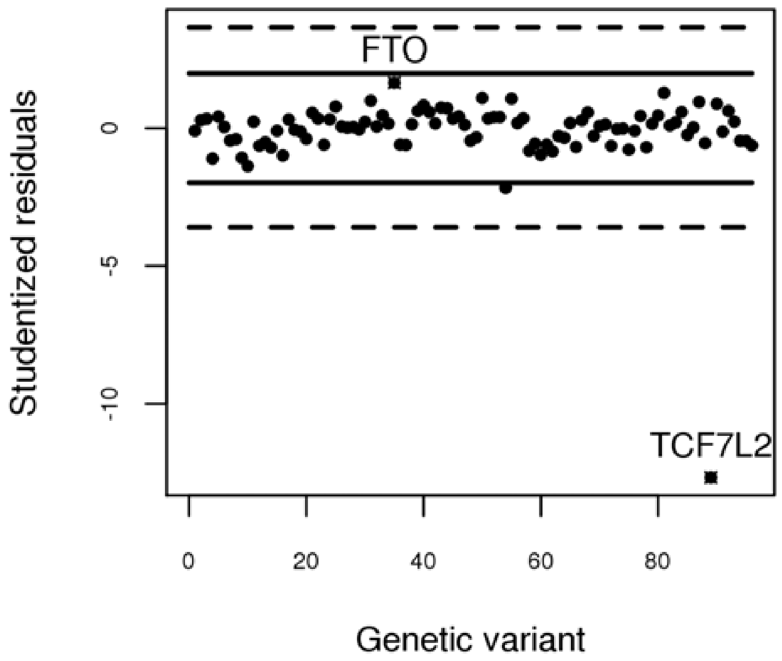

Example: Outlier analysis – Cook’s distance

“Figure S2A - Cook’s distance applied to the IVW method.” [9]

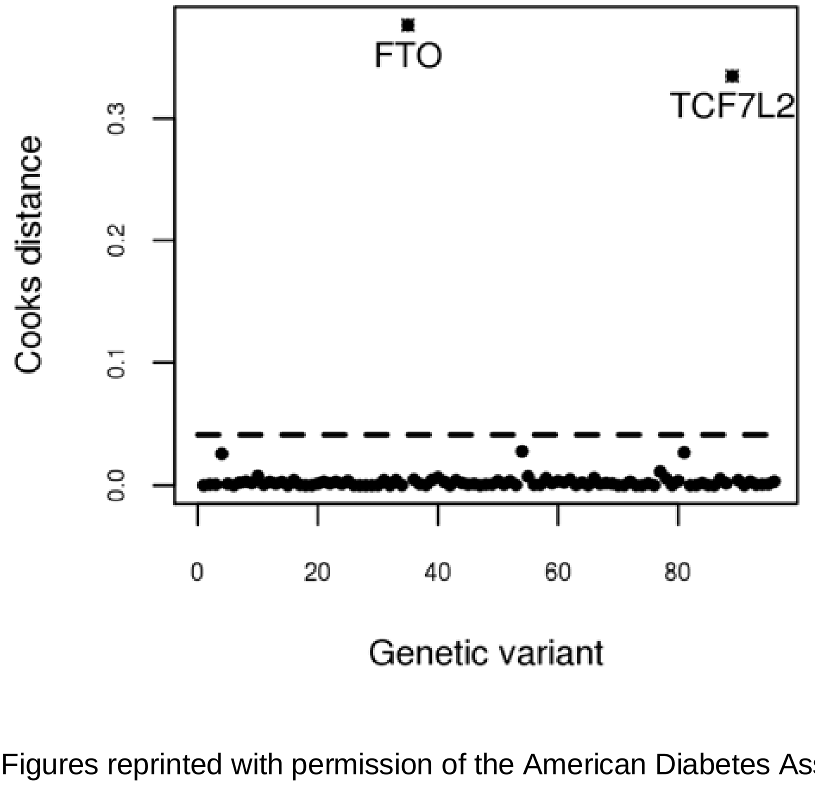

Figures reprinted with permission of the American Diabetes Association. Copyright 2016.

Limitations (item 15)

Discuss limitations of the study, taking into account the validity of the IV assumptions, other sources of potential bias, and imprecision. Discuss both direction and magnitude of any potential bias and your efforts to address them.

Example: “Since the prevalence of counseling increases with increasing levels of obesity, our estimates may overestimate the true prevalence. Telephone surveys also may overestimate the true prevalence of counseling. Although persons without telephones have similar levels of overweight as persons with telephones, persons without telephones tend to be less educated, a factor associated with lower levels of counseling in our study. Also, of concern is the potential bias caused by those who refused to participate as well as those who refused to respond to questions about weight. Furthermore, because the data were collected cross-sectionally, we cannot infer that counseling preceded a patient's attempt to lose weight.” [10]

References

1. Allin KH, Nordestgaard BG, Zacho J, Tybjærg-Hansen A, Bojesen SE. C-Reactive Protein and the Risk of Cancer: A Mendelian Randomization Study. JNCI J Natl Cancer Inst. 2010;102(3):202–6.

2. Carreras-Torres R, Johansson M, Haycock PC, Relton CL, Davey Smith G, Brennan P, et al. Role of obesity in smoking behaviour: Mendelian randomisation study in UK Biobank. BMJ. 2018;361(NA):k1767.

3. Trajanoska K, Morris JA, Oei L, Zheng H-F, Evans DM, Kiel DP, et al. Assessment of the genetic and clinical determinants of fracture risk: genome wide association and mendelian randomisation study. BMJ. 2018;362:k3225.

4. Mhatre S, Richmond RC, Chatterjee N, Rajaraman P, Wang Z, Zhang H, et al. The Role of Gallstones in Gallbladder Cancer in India: A Mendelian Randomization Study. Cancer Epidemiol Biomarkers & Prev [Internet]. 2021 Feb 1;30(2):396 LP – 403. Available from: http://cebp.aacrjournals.org/content/30/2/396.abstract

5. Lotta LA, Wittemans LBL, Zuber V, Stewart ID, Sharp SJ, Luan J, et al. Association of Genetic Variants Related to Gluteofemoral vs Abdominal Fat Distribution With Type 2 Diabetes, Coronary Disease, and Cardiovascular Risk Factors. JAMA. 2018;320(24):2553–63.

6. Wade KH, Carslake D, Sattar N, Davey Smith G, Timpson NJ. BMI and Mortality in UK Biobank: Revised Estimates Using Mendelian Randomization. Obesity. 2018;26(11):1796–806.

7. Carter AR, Gill D, Davies NM, Taylor AE, Tillmann T, Vaucher J, et al. Understanding the consequences of education inequality on cardiovascular disease: mendelian randomisation study. BMJ. 2019;365:l1855.

8. Bowden J, Spiller W, Del Greco M F, Sheehan N, Thompson J, Minelli C, et al. Improving the visualization, interpretation and analysis of two-sample summary data Mendelian randomization via the Radial plot and Radial regression. Int J Epidemiol. 2018;47(4):1264–78.

9. Corbin LJ, Richmond RC, Wade KH, Burgess S, Bowden J, Davey Smith G, et al. BMI as a Modifiable Risk Factor for Type 2 Diabetes: Refining and Understanding Causal Estimates Using Mendelian Randomization. Diabetes. 2016;65(10):3002.

10. Galuska DA, Will JC, Serdula MK, Ford ES. Are Health Care Professionals Advising Obese Patients to Lose Weight? JAMA. 1999;282(16):1576–8.
